# Disparate dynamics of pathogen prevalence in *Ixodes ricinus* and *Dermacentor reticulatus* ticks occurring sympatrically in diverse habitats

**DOI:** 10.1038/s41598-023-37748-z

**Published:** 2023-06-30

**Authors:** Zbigniew Zając, Dasiel Obregon, Angélique Foucault-Simonin, Alejandra Wu-Chuang, Sara Moutailler, Clemence Galon, Joanna Kulisz, Aneta Woźniak, Katarzyna Bartosik, Alejandro Cabezas-Cruz

**Affiliations:** 1 Department of Biology and Parasitology, Medical University of Lublin, Radziwiłłowska 11 St, 20-080 Lublin, Poland; 2 School of Environmental Sciences University of Guelph, Guelph, ON N1G 2W1 Canada; 3 Anses, INRAE, Ecole Nationale Vétérinaire d’Alfort, UMR BIPAR, Laboratoire de Santé Animale, 94700 Maisons-Alfort, France

**Keywords:** Ecology, Ecological epidemiology, Microbial ecology, Molecular ecology

## Abstract

*Ixodes ricinus* and *Dermacentor reticulatus* ticks are important reservoirs and vectors of pathogens. The aim of the present study was to investigate the dynamic of the prevalence and genetic diversity of microorganisms detected in these tick species collected from two ecologically diverse biotopes undergoing disparate long-term climate condition. High-throughput real time PCR confirmed high prevalence of microorganisms detected in sympatrically occurring ticks species. *D. reticulatus* specimens were the most often infected with *Francisella*-like endosymbiont (FLE) (up to 100.0%) and *Rickettsia* spp. (up to 91.7%), while in case of *I. ricinus* the prevalence of Borreliaceae spirochetes reached up to 25.0%. Moreover, pathogens belonging to genera of *Bartonella*, *Anaplasma*, *Ehrlichia* and *Babesia* were detected in both tick species regardless the biotope. On the other hand, *Neoehrlichia mikurensis* was conformed only in *I. ricinus* in the forest biotope, while genetic material of *Theileria* spp. was found only in *D. reticulatus* collected from the meadow. Our study confirmed significant impact of biotope type on prevalence of representatives of Borreliaceae and Rickettsiaceae families. The most common co-infection detected in *D. reticulatus* was *Rickettsia* spp. + FLE, while Borreliaceae + *R. helvetica* was the most common in *I. ricinus*. Additionally, we found significant genetic diversity of *R. raoultii gltA* gene across studied years, however such relationship was not observed in ticks from studied biotopes. Our results suggest that ecological type of biotope undergoing disparate long-term climate conditions have an impact on prevalence of tick-borne pathogens in adult *D. reticulatus* and *I. ricinus*.

## Introduction

*Ixodes ricinus* and *Dermacentor reticulatus* are two of the most widely distributed representatives of over 60 species of ticks (Acari: Ixodida) occurring in the Western Palearctic^1^. In Europe, *I. ricinus* is common throughout the continent^2^, while the compact geographical range of *D. reticulatus* extends from the western parts of the British Isles and northern Italy through the countries of Central Europe to the Ural Mountains in the east^3^.

The habitats preferred by *I. ricinus* include deciduous and mixed forests, pastures, and moorlands. Specimens of this species are often found in city parks as well^2, 4^*. D. reticulatus* ticks are most frequently collected in meadow habitats and river valleys but less frequently in forest habitats^5–7^. Populations of *D. reticulatus* were found to be particularly abundant in habitats of agriculturally unused fields, especially those undergoing progressive ecological succession^8, 9^.

With their wide occurrence range, considerable population size, plasticity in relation to occupied habitats, and diverse range of host organisms, *I. ricinus* and *D. reticulatus* ticks play an important role in the transmission and maintenance of tick-borne pathogen foci in the environment^10^. In the case of pathogens transmitted by *I. ricinus*, the highest medical importance is ascribed to *Borrelia burgdorferi* sensu lato (s.l.) spirochetes, which cause Lyme borreliosis (LB) in humans, and tick-borne encephalitis virus (TBEV), i.e., the etiological agent of tick-borne encephalitis in humans (TBE)^11^. Besides mentioned pathogens *I. ricinus* ticks are reservoirs and vectors of e.g., *Babesia* sp. *Bartonella* sp*., Neoehrlichia mikurensis* and microorganisms representing *Francisella* sp. These pathogens are commonly detected in ticks from previous reports, affecting human and animal health and may cause diagnostic difficulties^12–14^.

*D. reticulatus* ticks attack humans sporadically but can transmit TBEV^15, 16^ as well as *Rickettsia slovaca* and *R. raoultii*, which are agents of tick-borne lymphadenopathy (TIBOLA)^17^. The ability of *D. reticulatus* to transmit *B. burgdorferi* s.l., and *Francisella tularensis* has not been fully elucidated^12, 18^. In veterinary practice, the most important pathogens transmitted by *D. reticulatus* are *Babesia canis* protozoa causing canine babesiosis and *Anaplasma marginale* rickettsiae responsible for the development of bovine anaplasmosis^19–21^.

According to predictions, progressive climate change will affect the distribution ranges of various tick species and their hosts, changing the incidence of tick-borne diseases and zoonotic disease risk^22^. Ecological networks analysis suggested that interactions between ticks and transmitted pathogens evolved to minimise interspecific competition while allowing ample but modular circulation of transmitted pathogens among vertebrates^23^. Similarly, ecological network interactions between *I. ricinus* ticks and their hosts evolved to maximize habitat overlap with some hosts that are super-spreaders of pathogens^24^. Despite tick density being highly influenced by the presence of suitable hosts^22^, immediate tick survival and questing activities are highly dependent on suitable and specific environmental conditions (temperatures between 8 and 24 °C and humidity of up to 80%). In addition to environmental variables and host availability, shifts in tick microbiota dynamics over time were also reported to influence the presence of tick-borne pathogens (TBPs) in ticks^25^. The complex interactions between abiotic and biotic factors can cause temporal variations in tick-borne pathogen prevalence^26^. For instance, significant variations in seasonal and/or inter-annual pathogen prevalence were observed for *R. helvetica*, *B. burgdorferi* s.l., *B. miyamotoi* and *A. phagocytophilum* in *I. ricinus* over a period of three years in a French peri-urban forest^26^. Understanding how variations in microclimate conditions impact tick-borne pathogen prevalence in questing ticks is important for predictions of zoonotic disease risk under global climatic fluctuations^27, 28^.

In this study we collected questing adult *I. ricinus* and *D. reticulatus* ticks over a period of three years in two biotopes (the forest and the meadow) with differences in climate condition and we assessed variations in prevalence of multiple tick-borne pathogens and endosymbiont (hereafter microorganisms) across time, as well as between biotopes and tick species. We additionally compared the occurrence of co-infections of TBPs and genetic diversity of pathogens of the same species between sites and tick species. Hence, we hypothesized possible differences in species diversity, prevalence and genetic diversity of detected TBPs in sympatric tick species collected from ecologically and climatically diverse habitats.

## Results

### Selecting biotope locations for assessing pathogen prevalence using long-term climatic data

The two regions within which study transects were established differed significantly in long-term climatic data, i.e., mean temperature (t = − 2.11, *p* = 0.02); total sum of precipitation (t = 3.20, *p* < 0.01); length of vegetation period (t = 18.39, *p* < 0.01), in opposite to total number of days with snow cover (t = 0.52, *p* = 0.30).

### Population dynamics of *Dermacentor reticulatus *and *Ixodes ricinus* in diverse habitats

**Figure 1:**
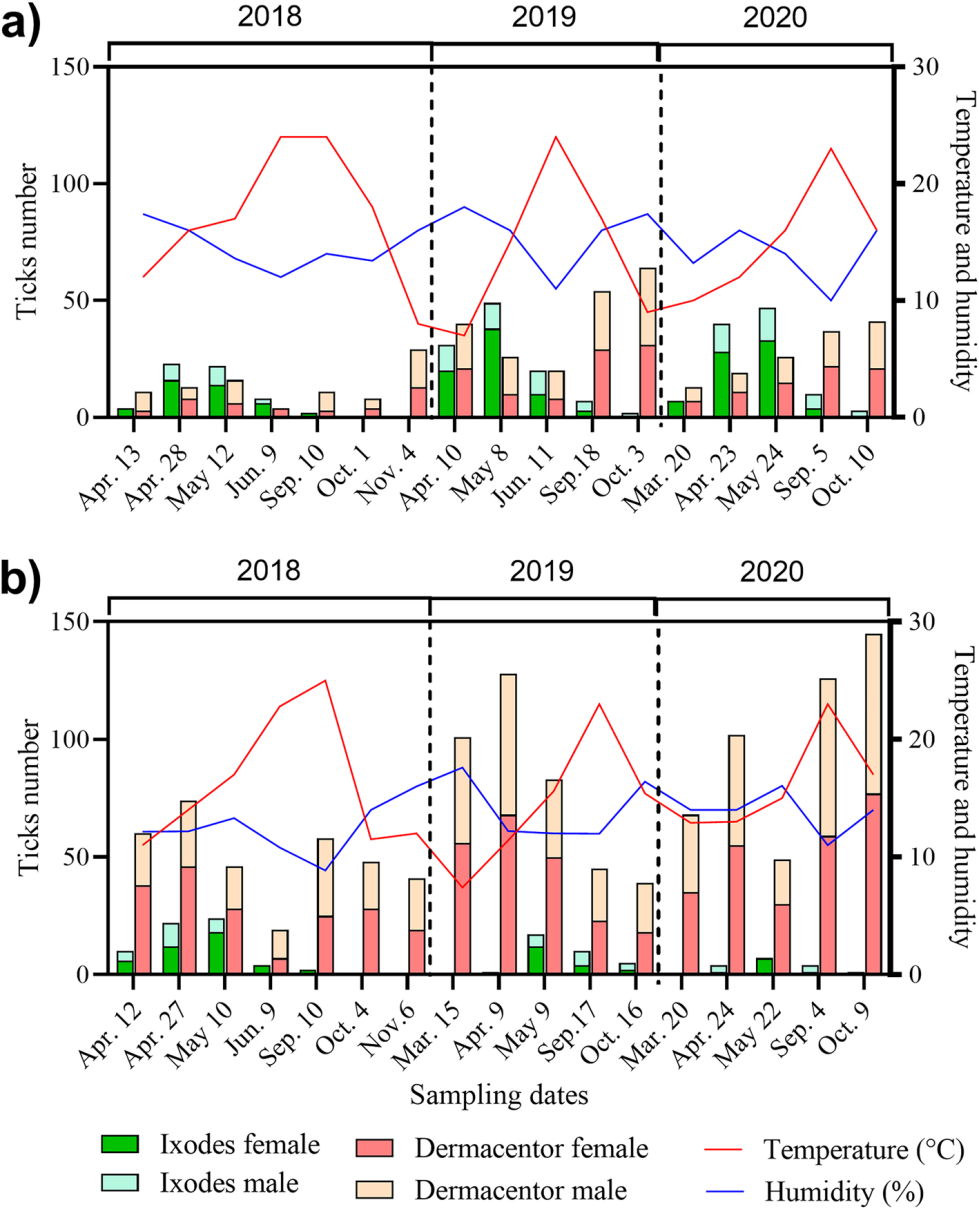
Population dynamics of questing *I. ricinus* and *D. reticulatus* ticks in two ecological sites. (**a**) and (**b**) indicate the studied forest and meadow biotopes, respectively. Tick counts were differentiated between females and males from each tick species. The measurements were conducted for three consecutive years. The seasonal variation of environmental temperature and humidity were measured and displayed.

Two tick species *D. reticulatus* and *I. ricinus* were found at different frequencies in the two biotopes included in the study. In total, 1664 adults of *D. reticulatus* and 386 of *I. ricinus* ticks were collected in the forest biotope (*D. reticulatus n* = 432, *I. ricinus n* = 275) and meadow biotope (*D. reticulatus n* = 1,232; *I. ricinus n* = 111) over three years (2018–2020). *D. reticulatus* was dominant in both biotopes (Fig. 1). The abundance of *D. reticulatus* in the meadow biotope was significantly higher than in the forest biotope (Z = − 4.06, *p* < 0.01), while the abundance of *I. ricinus* was not statistically different between biotopes (Z = 1.72, *p* = 0.09). Two peaks of *D. reticulatus* activity were observed each year, in spring and in autumn, whereas *I. ricinus* population peaked only once a year, in spring (Fig. 1). The studied biotopes did not differ significantly in terms of values of temperature (H = 0.44, *p* = 0.66) and humidity (H = 1.80, *p* = 0.07) at time of tick collection. Moreover, no significant correlations were found between temperature or humidity and the number of active *I. ricinus* or *D. reticulatus* ticks (Supplementary Fig. S1).

### Prevalence of microorganisms in *Ixodes ricinus* and *Dermacentor reticulatus* ticks

**Figure 2:**
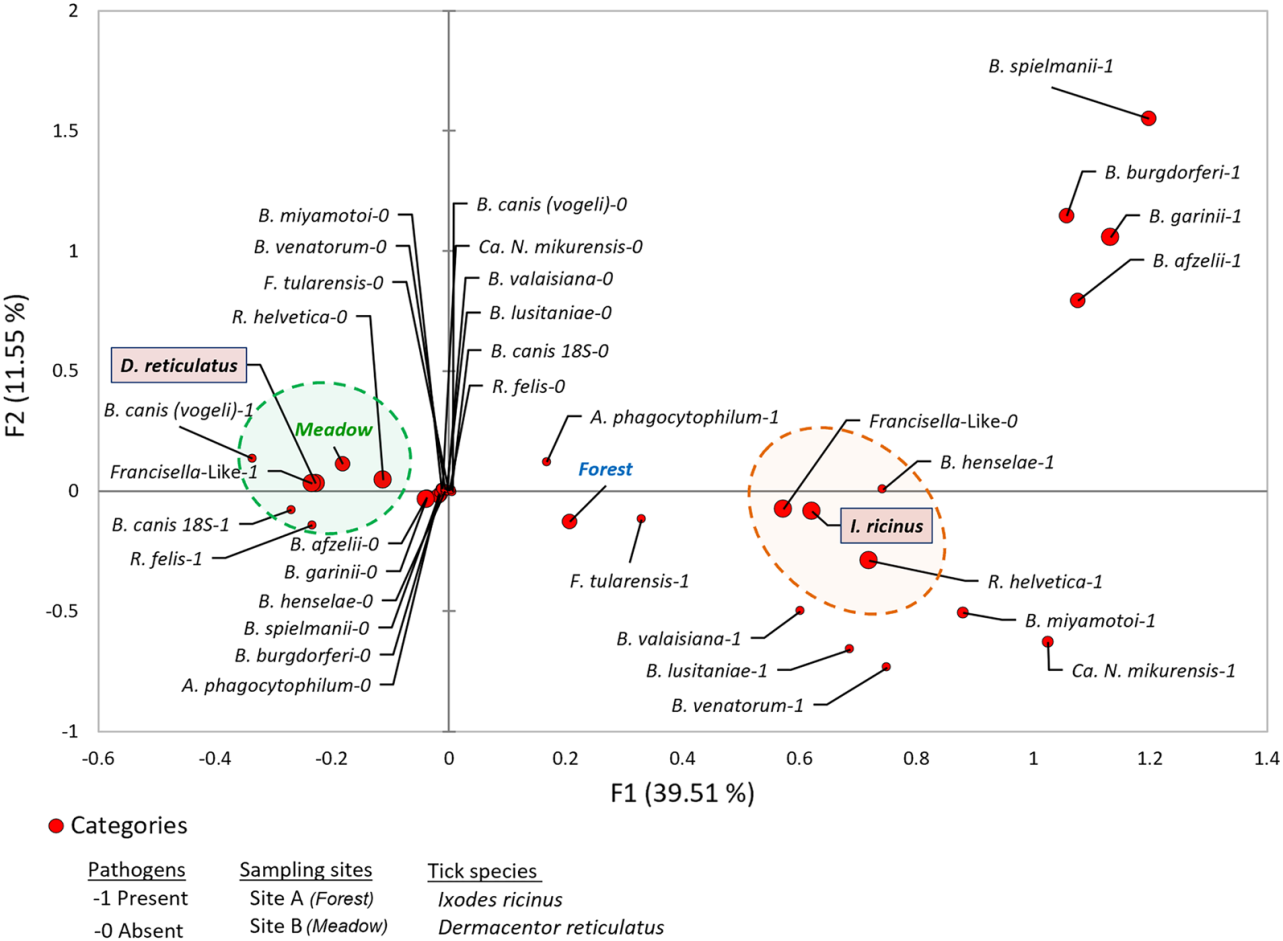
Multiple correspondence analysis (MCA) plot showing associations between the tick-borne pathogens, tick species and sampling sites in Poland. The presence or absence of the pathogens is denoted by 1 and 0, respectively. The ellipsoid encompasses groups of pathogens associated with each sampling forest biotope end/or tick species. Node sizes are proportional to the pathogen prevalence in ticks. The fraction of each component is represented as a percentage for each dimension (axis).

High-throughput screening of TBPs and one endosymbiont (*Francisella*-like endosymbiont–FLE) DNA was carried out in questing ticks collected in the forest (*D. reticulatus n* = 81, *I. ricinus n* = 150; Table 1) and meadow (*D. reticulatus* n = 177, *I. ricinus* n = 35; Table 2) biotopes. The examined tick species differed in the number of infections by different tick-borne microorganisms. *I. ricinus* ticks had a greater variety of microorganisms (i.e., number of different genera and/or species detected by tick specimen) than *D. reticulatus*, however *D. reticulatus* ticks were more often infected (Tables 1 and 2). A higher diversity of microorganisms was found in ticks collected in the forest biotope (17 microorganisms species detected in *I. ricinus* and 6 species detected in *D. reticulatus*), while in the meadow biotope 14 microbial species were detected in *I. ricinus* and 11 in *D. reticulatus* (Tables 1 and 2). A strong association, that was found with multiple correspondence analysis (MCA), between biotopes, ticks and microorganisms was observed for *D. reticulatus* and *B. canis,* FLE and *R. helvetica* in the meadow biotope (Fig. 2). *I. ricinus* showed a strong association with *R. helvetica* and *Bartonella henselae*, but not with any habitat in particular (Fig. 2). Other detected TBPs were not associated with either ecological sites or particular tick species, but different prevalence of individual microorganisms was found between sites and tick species (Fig. 2, Tables 1 and 2).

**Table 1:** Prevalence of microorganisms detected in *Ixodes ricinus* and *Dermacentor reticulatus* collected in forest biotope.

Year	Tick species		Number of infected specimens with particular microorganism including percentage rate (%)																
		*Rickettsia* spp*.*	*Rickettsia monacensis*	*Rickettsia helvetica*	*Borreliella* spp.	*Borreliella garinii*	*Borreliella spielmanii*	*Borreliella afzelii*	*Borreliella valaisiana*	*Borrelia miyamotoi*	*Borreliella lusitaniae*	*Bartonella henselae*	*Anaplasma phagocytophilum*	*Ehrlichia* spp.	*Francisella*-like endosymbiont	*Babesia canis*	*Babesia venatorum*	Apicomplexa^1^	*Neoehrlichia mikurensis*
2018	*I. ricinus* *n* = 20	3 (15.0)	1 (5.0)	7 (35.0)	2 (10.0)	5 (25.0)	1 (5.00)	4 (20.0)	0 (0.0)	1 (5.00)	0 (0.0)	2 (10.0)	5 (25.0)	2 (10.0)	0 (0.0)	0 (0.0)	0 (0.0)	3 (15.0)	1 (5.0)
	*D. reticulatus* *n* = 24	22 (91.7)	0 (0.0)	0 (0.0)	0 (0.0)	0 (0.0)	0 (0.0)	0 (0.0)	0 (0.0)	0 (0.0)	0 (0.0)	0 (0.0)	0 (0.0)	0 (0.0)	24 (100.0)	3 (12.5)	0 (0.0)	8 (33.3)	0 (0.0)
2019	*I. ricinus* *n* = 64	5 (7.8)	0 (0.0)	23 (36.0)	3 (4.7)	0 (0.0)	0 (0.0)	3 (4.7)	0 (0.0)	1 (1.5)	2 (3.1)	0 (0.0)	5 (7.8)	4 (6.2)	0 (0.0)	0 (0.0)	2 (3.1)	7 (11.0)	3 (4.7)
	*D. reticulatus* *n* = 35	30 (85.7)	0 (0.0)	0 (0.0)	0 (0.0)	0 (0.0)	0 (0.0)	0 (0.0)	0 (0.0)	0 (0.0)	0 (0.0)	0 (0.0)	3 (8.6)	0 (0.0)	35 (100.0)	0 (0.0)	0 (0.0)	7 (20.0)	0 (0.0)
2020	*I. ricinus* *n* = 66	0 (0.0)	0 (0.0)	25 (37.9)	2 (3.0)	0 (0.0)	1 (1.5)	4 (6.1)	1 (1.5)	2 (3.0)	0 (0.0)	0 (0.0)	3 (4.5)	1 (1.5)	0 (0.0)	3 (8.6)	3 (4.5)	9 (13.6)	1 (1.5)
	*D. reticulatus* *n* = 22	15 (68.2)	0 (0.0)	2 (9.1)	0 (0.0)	0 (0.0)	0 (0.0)	0 (0.0)	0 (0.0)	0 (0.0)	0 (0.0)	0 (0.0)	0 (0.0)	0 (0.0)	21 (95.4)	0 (0.0)	0 (0.0)	4 (18.2)	0 (0.0)

^1^Excluding *Babesia* spp. and *Theileria* spp., *n*—number of examined ticks.

**Table 2:** Prevalence of microorganisms detected in *Ixodes ricinus* and *Dermacentor reticulatus* collected in meadow biotope.

Year	Tick species	Number of infected specimens with particular microorganism including percentage rate (%)																	
		*Rickettsia* spp*.*	*Rickettsia raoultii*	*Rickettsia aeschlimannii*	*Bartonella* spp.	*Borreliella* spp.	*Borreliella burgdorferi* s.s	*Borreliella garinii*	*Borreliella spielmanii*	*Borreliella afzelii*	*Borreliella valaisiana*	*Borrelia miyamotoi*	*Bartonella henselae*	*Anaplasma phagocytophilum*	*Ehrlichia* spp.	*Francisella*-like endosymbiont	*Babesia canis*	*Theileria* spp.	Apicomplexa^1^
2018	*I. ricinus* *n* = *15*	2 (13.3)	0 (0.0)	0 (0.0)	0 (0.0)	0 (0.0)	2 (13.)	3 (20.0)	2 (13.3)	1 (6.7)	0 (0.0)	0 (0.0)	0 (0.0)	4 (26.7)	0 (0.0)	1 (6.7)	0 (0.0)	0 (0.0)	3 (20.0)
	*D. reticulatus* *n* = 47	25 (53.2)	3 (6.4)	1 (2.1)	0 (0.0)	0 (0.0)	0 (0.0)	0 (0.0)	0 (0.0)	1 (2.1)	0 (0.0)	0 (0.0)	0 (0.0)	6 (12.8)	0 (0.0)	46 (97.8)	2 (4.3)	1 (2.1)	9 (19.1)
2019	*I. ricinus* *n* = 12	0 (0.0)	0 (0.0)	0 (0.0)	2 (16.7)	2 (16.7)	2 (16.7)	0 (0.0)	0 (0.0)	0 (0.0)	1 (8.3)	1 (8.3)	0 (0.0)	1 (8.3)	1 (8.3)	0 (0.0)	0 (0.0)	0 (0.0)	0 (0.0)
	*D. reticulatus* *n* = *72*	47 (65.3)	4 (5.6)	0 (0.0)	0 (0.0)	0 (0.0)	0 (0.0)	0 (0.0)	0 (0.0)	0 (0.0)	0 (0.0)	0 (0.0)	0 (0.0)	4 (5.6)	0 (0.0)	62 (86.1)	2 (2.8)	0 (0.0)	10 (13.9)
2020	*I. ricinus* *n* = 8	1 (12.5)	0 (0.0)	0 (0.0)	0 (0.0)	1 (12.5)	0 (0.0)	1 (12.5)	1 (12.5)	1 (12.5)	0 (0.0)	0 (0.0)	1 (12.5)	0 (0.0)	0 (0.0)	0 (0.0)	0 (0.0)	0 (0.0)	0 (0.0)
	*D. reticulatus* *n* = *58*	44 (75.6)	2 (3.4)	0 (0.0)	2 (3.4)	0 (0.0)	0 (0.0)	0 (0.0)	0 (0.0)	0 (0.0)	0 (0.0)	0 (0.0)	0 (0.0)	5 (8.6)	1 (1.7)	55 (94.8)	2 (3.4)	0 (0.0)	8 (13.8)

^1^Excluding *Babesia* spp. and *Theileria* spp.,* n*-number of examined ticks.

### Prevalence of *Rickettsia* spp

The presence of genetic material of *Rickettsia* spp. was confirmed in specimens from both tick species. In the forest biotope, *R. helvetica* and uncharacterized *Rickettsia* spp. were detected in *D. reticulatus* ticks, while *I. ricinus* ticks were infected by *R. monacensis*, *R. helvetica* and also by uncharacterized *Rickettsia* spp. (Table 1). In the meadow biotope, *D. reticulatus* ticks were infected by *R. raoultii*, *R. aeschlimannii* and uncharacterized *Rickettsia* spp.; while only uncharacterized *Rickettsia* spp. were found in *I. ricinus* (Table 2). There was a significant difference in *Rickettsia* prevalence between the sites regardless of the tick species (χ^2^ = 7.18, *p* = 0.02).

A higher prevalence of *Rickettsia* bacterial infection was found in *D. reticulatus*, compared to *I. ricinus* in both sites (χ^2^ = 9.32, *p* = 0.01). In the forest biotope, *Rickettsia* prevalence varied yearly from 68.2 to 91.7% in *D. reticulatus* depending on the year, but the differences in prevalence across time were not significant (χ^2^ = 4.81,* p* = 0.09). In the case of *I. ricinus* ticks from the forest biotope, the prevalence of *Rickettsia* ranged from 5.0 to 37.9%, and no significant differences were found between the consecutive years of the study (χ^2^ = 5.42, *p* = 0.06) (Table 1). In the meadow biotope, rickettsial infection was confirmed in up to 75.6% of *D. reticulatus* and in up to 13.3% of *I. ricinus* specimens (Table 2). The differences of prevalence of *Rickettsia* infection across the time was statistically significant for both tick species, i.e., χ^2^ = 13.70, *p* < 0.01; χ^2^ = 10.57, *p* < 0.01, respectively.

### Prevalence of spirochetes of the Borreliaceae family

Genetic material of spirochetes of the Borreliaceae family (i.e., *Borreliella garinii, B. spielmanii, B. afzelii, B. valaisiana, B. lusitaniae,* as well as *Borrelia miyamotoi*) was detected mainly in *I. ricinus* collected in the forest biotope. *B. afzelii* infection was also found in one *D. reticulatus* individual from the meadow biotope (Table 2).

The prevalence of Borreliaceae spirochetes ranged from 1.5 to 25.0% in the forest biotope (Table 1), and from 2.1 to 20.0% in the meadow biotope (Table 2). The prevalence of Borreliaceae differed significantly in *I. ricinus* populations from the forest and the meadow biotopes (χ^2^ = 27.81, *p* < 0.01). The prevalence of Borreliaceae-infected *I. ricinus* ticks varied across time in the forest (χ^2^ = 79.04, *p* < 0.01), but not in the meadow biotope (χ^2^ = 3.31, *p* = 0.19).

### Prevalence of *Anaplasma phagocytophilum*

In *I. ricinus*, the prevalence of *A. phagocytophilum* infections ranged from 4.5% in ticks collected from the forest biotope up to 26.7% in the meadow biotope (Tables 1 and 2). The presence of *A*. *phagocytophilum* in *D. reticulatus* inhabiting the forest biotope was confirmed only in 2019 year (8.6%), while in the meadow biotope infected specimens were collected in all studied years (up to 12.8%; Tables 1, 2). The prevalence of *A. phagocytophilum* infection varied significantly between tick species, regardless of biotope (ticks collected in the forest biotope χ^2^ = 20.39, *p* < 0.01; in the meadow biotope χ^2^ = 15.90, *p* = 0.03) (Tables 1, 2). No differences in the prevalence of this pathogen were noted between the biotopes (χ^2^ = 0.04, *p* = 0.94); however, temporal patterns of *A. phagocytophilum* infection were significantly different in the forest (χ^2^ = 21.03, *p* < 0.01) and the meadow biotope (χ^2^ = 12.50, *p* = 0.04) (Tables 1 and 2).

### Prevalence of *Babesia* spp

In the forest biotope, genetic material of *B. canis* was detected in 12.5% of *D. reticulatus* ticks in 2018 and in 8.6% of *I. ricinus* ticks in 2020 (Table 1), while *B. venatorum* was confirmed in 3.1% of *I. ricinus* specimens in 2019 and 4.5% in 2020. In the meadow biotope, *B. canis* was found only in *D. reticulatus* ticks (up to 4.3% of infected specimens) (Table 2). There was no significant difference in the *Babesia* spp. prevalence between the biotopes (χ^2^ = 0.60, *p* = 0.74) and between studied years (χ^2^ = 3.26, *p* = 0.20).

### Prevalence of other tick-borne pathogens

Depending on the year, up to 16.7% of the ticks were infected by *Bartonella* spp. The presence of *Theileria* spp. was confirmed in one *D. reticulatus* specimen (2.1%) in the meadow biotope, while *N. mikurensis* was detected only in *I. ricinus* specimens collected in the forest biotope (up to 5.0%). From 11.0 to 33.3% of the ticks in both sites were infected by Apicomplexa parasites, excluding *Babesia* spp. or *Theileria* spp. (Tables 1 and 2).

### Prevalence of tick *Francisella*-like endosymbiont (FLE)

In both the forest and meadow biotopes high prevalence of FLE was found only in *D. reticulatus,* reaching from 95.4 to 100.0% and from 86.1 to 97.8%, respectively (Tables 1 and 2). Whereas in the case of *I. ricinus* only one specimen collected in the meadow biotope was found FLE-positive (Tables 1 and 2). Significant differences were observed in the prevalence of FLE between tick species at both the forest (*p* < 0.01) and the meadow biotope (χ^2^ = 12.49, *p* < 0.01), but there were no significant differences in FLE prevalence between the study years (χ^2^ = 0.62, *p* = 0.73).

### Co-infections in *Ixodes ricinus* and *Dermacentor reticulatus* ticks

**Figure 3:**
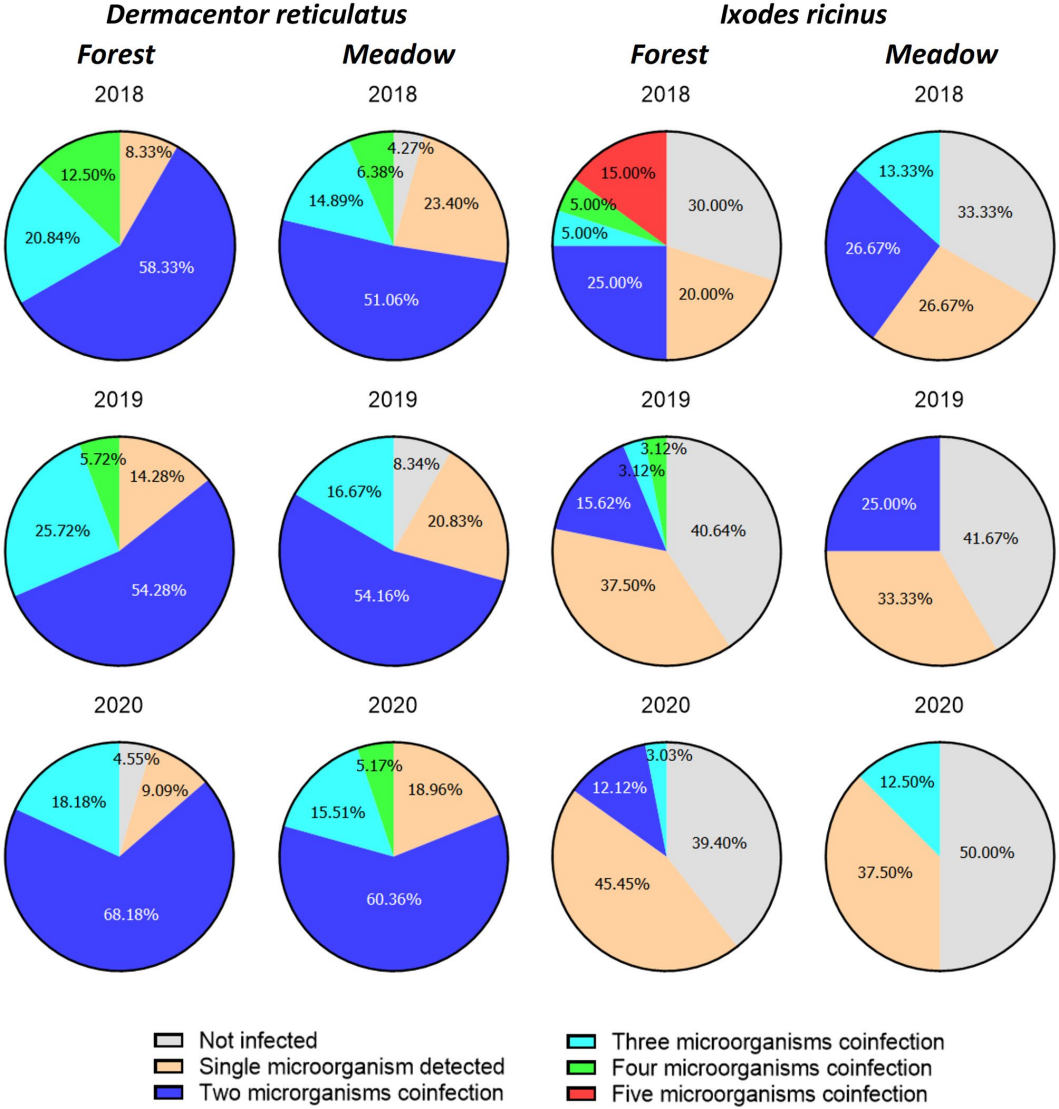
Prevalence of microorganism co-infection in *I. ricinus* and *D. reticulatus* ticks collected from examined sites in 2018–2020.

The analyzed populations of *I. ricinus* and *D. reticulatus* exhibited a high rate of co-infections by tick-borne microorganisms (Fig. 3; Supplementary Table S1). The coexistence of multiple microorganisms was observed more frequently in *D. reticulatus*. In the study years, 85.72–100.00% of all *D. reticulatus* ticks were infected by two or more pathogens (Fig. 3). The most common co-infections were *Rickettsia* spp*.* + FLE and *Rickettsia* spp. + FLE + *B. canis* (Supplementary Table S1). There was no significant difference in the prevalence of polymicrobial infections in *D. reticulatus* between habitats (χ^2^ = 0.47, *p* = 0.78).

In *I. ricinus* single infections were frequently observed (ranged from 20.00 to 45.45%) and 30.00–50.00% of ticks of this species were not infected by any of the microorganisms (Fig. 3). Additionally, co-infections by up to five microorganisms were detected in the forest biotope and infections by maximum of three microorganisms were found in the meadow biotope (Fig. 3). The most common co-infecting pathogens in *I. ricinus* were Borreliacea + *R. helvetica* (Supplementary Table S1). However, no statistically significant difference in the prevalence of co-infections in *I. ricinus* was found between biotopes (χ^2^ = 0.51, *p* = 0.77).

In the case of *I. ricinus*, a reduction in co-infections with two or more microorganisms was observed over the studied years. This relationship was observed regardless of the type of biotope, i.e., the forest biotope: χ^2^ = 30.89, *p* < 0.01; the meadow biotope χ^2^ = 20.07, *p* < 0.01. On the other hand, the level of multiple co-infections in *D. reticulatus*, remained stable across the study period, with no significant variations (χ^2^ = 0.46, *p* = 0.79) (Fig. 3).

### Phylogenetic and genetic diversity of microorganisms detected in *Ixodes ricinus* and *Dermacentor reticulatus*

**Figure 4:**
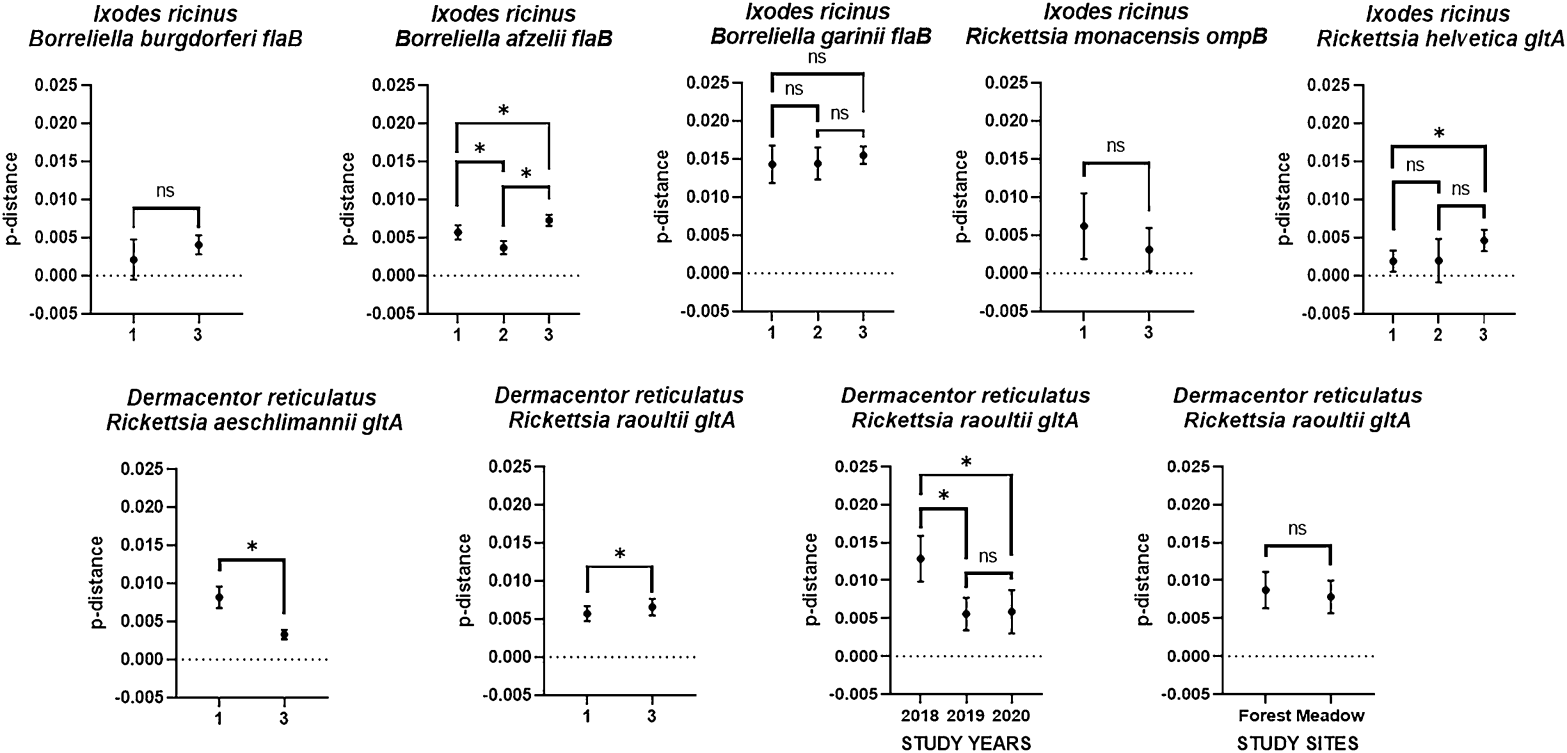
Genetic diversity of tick-borne pathogens detected in *I. ricinus* and *D. reticulatus* in current study. 1-sequences from current study, 2-sequences form other studies from Poland, 3-sequences from other countries.

Phylogenetic analysis of the *flaB* gene sequences in *B. burgdorferi* sensu stricto (s.s.). (OQ254784) showed that these sequences clustered with another sequence from Japan (Supplementary Fig. S2) and were not significantly different compared to all other sequences included in the phylogenetic tree (Fig. 4). In the result of Maximum Likelihood analysis, *B. afzelii flaB* (OQ076388, OQ076395) clustered together with other sequences reported from previous studies in Poland (Supplementary Fig. S3); however, they differed significantly in case of the proportion of nucleotide sites changes (Fig. 4, Table 3). *B. garinii flaB* (OQ076404, OQ076403) were clustered with other sequences from Poland and Europe with no significant differences in p-distance (Fig. 4, Supplementary Fig. S4, Table 3).

**Table 3:** Statistical differences in changes of nucleotide position in isolated genes.

Tick species	Pathogen species and target gene	*p* values		
		Compared groups		
		1 and 2	1 and 3	2 and 3
*Ixodes ricinus*	*Borreliella burgdorferi flaB*	nd	0.22	nd
	*Borreliella afzelii flaB*	0.02	< 0.01	< 0.01
	*Borreliella garinii flaB*	0.94	0.37	0.32
	*Rickettsia monacensis ompB*	nd	0.28	nd
	*Rickettsia helvetica gltA*	> 0.99	< 0.01	0.26
*Dermacentor reticulatus*	*Rickettsia aeschlimannii gltA*	nd	< 0.01	nd
	*Rickettsia raoultii gltA*	nd	< 0.01	nd

1-current study; 2-other studies from Poland; 3-studies from other countries; nd-no data, no statistical calculations performed; *p*-level of significance.

*R. monacensis ompB* gene isolated from *I. ricinus* in present study (OQ076408) was clustered with other sequences from Europe and the proportion of nucleotide sites changes between them were not significant (Fig. 4, Supplementary Fig. S5, Table 3). Similarly, there was no significant differences in p-distance between current study and other sequences from Poland in case of *R. helvetica gltA* (OQ102267-71) (Fig. 4, Supplementary Fig. S6, Table 3). Statistically significant differences of the proportion of nucleotide sites changes between sequences obtained in present study and those reported from other countries were observed in case of *R. aeschlimannii gltA* (OQ102275) and *R. raoultii gltA* (OQ102285-88) isolated from *D. reticulatus* (Fig. 4, Supplementary Figs. S7, S8, Table 3).

Analysis of genetic diversity of *R. raoultii gltA* sequences (Supplementary Fig. S9) from our study showed statistically insignificant differences of p-distance between the forest and the meadow biotopes (*p* = 0.64); however significant differences were observed between studied years of 2018 and 2019 (*p* < 0.01), 2018 and 2020 (*p* < 0.01) (Fig. 4, Table 3).

## Discussion

Our results confirmed the occurrence of ticks and the presence of a broad spectrum of microorganisms in adults of *I. ricinus* and *D. reticulatus* regardless habitat type. Similar observations were reported from other previous studies from eastern Poland^29^. Although, *I. ricinus* and *D. reticulatus* prefer various habitat types where their abundance is the highest, both species may be found in the same ecosystems. This observation was confirmed in our study (Fig. 1; Tables 1 and 2). We observed lack of significant differences of number of *I. ricinus* ticks collected from studied biotopes (Fig. 1). In our opinion it is caused by relatively low abundance *of I. ricinus* collected from both habitats. Also, *I. ricinus* can be found in meadow habitats too^2, 4, 30^. In case of our study, occurrence of *I. ricinus* in the meadow biotope can also be explained by close vicinity of forest and location of the transect used in the study in the river valley which favors the maintenance of humidity in environment known to affect water balance in ticks, which is critical for this tick species^2, 30^. Co-occurrence of ticks can also favor transmission of pathogens between habitats and may support pathogens’ maintenance in the environment^31^. Based on the obtained results, it was observed that the studied sites were situated in subregions that exhibited variations in long-term climatic data. These differences in climatic conditions could potentially influence the vegetation and the range of hosts available for ticks. However, it is important to note that there were no significant differences observed in terms of microclimatic conditions such as air temperature and relative humidity (Fig. 1). This finding suggests that the occurrence of the same tick species and pathogens in both biotopes can be attributed to the similarities in microclimatic conditions^32^, rather than aforementioned variations in long-term climatic conditions.

Bacteria representing the genus *Rickettsia* were the most common pathogens detected in *D. reticulatus* ticks in the study sites. The presence of the genetic material of pathogens from this genus (various species) was confirmed up to in 91.7% of *D. reticulatus* ticks collected from the forest biotope in 2018 and in up to 75.6% of ticks from the meadow biotope in 2020. For comparison, *Rickettsia* spp. infected 15.0% of *I. ricinus* ticks from the forest biotope in 2018 (Tables 1 and 2). The study results indicate that the prevalence of *Rickettsia* spp. in *D. reticulatus* in the studied sites is higher than in other regions of the country. Previous studies on TBPs in eastern Poland reported that 53.0% of *D. reticulatus* ticks were infected by *Rickettsia* spp.^33^. The rate of *Rickettsia* sp. infections in *D. reticulatus* ticks was estimated at 41.0% in northern Poland^34^ and 57.0% in the northeastern part of the country^35^. A high prevalence of *Rickettsia* spp. infections in *D. reticulatus* was also reported in other Central and Eastern European countries^36, 37^. In the UK, in turn, Tijsse-Klasen et al.^38^ reported only 5.0% of *Rickettsia* sp. infection in *D. reticulatus* ticks. The high prevalence of *Rickettsia* sp. infections in ticks may be related to the fact that these pathogens can be regarded as endosymbionts of ticks^39^. It is also supported by the possibility of transovarial and transstadial transmission of *Rickettsia* pathogens^40^.

The present results show a significant difference in the prevalence of *Rickettsia* sp. between *I. ricinus* and *D. reticulatus*; nevertheless, the prevalence of infection of *I. ricinus* ticks in the analyzed sites should be regarded as high, in comparison with the results reported by other researchers, with minimum infection rate (MIR) reaching 8.6% of nymphs and adults of *I. ricinus* ticks collected in 2013 in Białowieża Primeval Forest^41^ (Tables 1 and 2). The relatively high prevalence of *Rickettsia* spp. in *I. ricinus* may contribute to the risk of transmission of these pathogens to humans. Research carried out in northeastern Poland has indicated the presence of anti-*Rickettsia* IgG antibodies in 39.02% of professionals that are most exposed to the risk of tick attacks (foresters and farmers)^42^.

From an epidemiological point of view, *Borreliella* spp. infections are the most important threat posed by TBPs to public health^43^. In the analyzed study sites, we observed a high prevalence of *Borreliella* spp. infection in *I. ricinus* ticks (depending on the study year, 1.5–25.0%) (Tables 1 and 2). Reports from other regions of the country show *Borrelia* spp. infection in 0.25% of *I. ricinus* ticks on the Baltic coast and 4.5% in the Opole region^13, 44^. Results reported by other authors confirm the trends observed in other Central European countries, where *B. afzelii* and *B. garinii* are the dominant *Borrelia* genospecies, similarly to the area analyzed in the present study^45^.

The *I. ricinus* populations analyzed in the current study differed significantly in the prevalence of Borreliacea spirochetes infections. This is most probably related to the differences in the ecological structure of the habitats where the ticks were collected and the spectrum of small rodent species inhabiting these sites^46^.

Similarly, to the present results (2.1%) (Tables 1 and 2), a low prevalence of *Borreliella* spp. in *D. reticulatus* was observed in previous studies conducted in eastern Poland and Europe^47, 48^. It is assumed that *Dermacentor* ticks are not competent vectors of *Borrelia* spp., as the development of spirochetes is inhibited by midgut secretions and the immune system of these ticks^49^.

As indicated by the results of the present study, *I. ricinus* ticks and, to a lesser extent, *D. reticulatus* should be regarded as important vectors and reservoirs of *A. phagocytophilum* in eastern Poland (Tables 1 and 2). There was no significant difference in the prevalence of *A. phagocytophilum* between the forest and the meadow biotopes, but the difference in the prevalence between the subsequent years turned out to be significant. This probably results from the variability of the structure of hosts for juvenile ticks in the habitat (periodic flooding). Differences in the *A. phagocytophilum* prevalence depending on *I. ricinus* and *D. reticulatus* habitats were reported from western Ukraine^50^.

In Europe, *D. reticulatus* is the main reservoir and vector of *B. canis*^17^. However, *I. ricinus* ticks also play an important role in the maintenance and circulation of this pathogen in nature^51^. *B. canis* and *Rickettsia* spp. co-infections^52^ are frequently observed in *D. reticulatus*, as confirmed by the results of the present study (Tables 1 and 2; Supplementary Table S1). In the macroregional scale, a higher prevalence of *B. canis* infections in *D. reticulatus* ticks has been observed in eastern regions of Europe. For example, the protozoan was found to infect up to 3.14% of examined tick specimens in Ukraine^53^. In turn, a lower percentage (0.3% to 1.64%) was reported from Western European countries^54^.

The present study confirmed *Bartonella* spp. infections in the ticks (up to 16.7%) (Tables 1 and 2). This indicates that the prevalence of infections by this bacterium in the analyzed sites is higher than e.g., in Ukraine (2.7–8.1%)^55^. Eastern Poland is an area with numerous populations of elks (*Alces alces*) and other deer ungulates (Cervidae) which may explain the high prevalence of *Bartonella* sp. in ticks^56^. These animals are one of the main reservoir hosts of *Bartonella.* Taking into account high tick infestation in deer, Cervidae can play important role in maintenance and circulation of *Bartonella* in environment^57, 58^. Our study also showed the presence of *N. mikurensis* in *I. ricinus* ticks collected in the forest biotope (Table 1). This bacterium is most often detected in *I. ricinus* from forest habitats and recreational areas^14, 59^.

The analyzed *I. ricinus* and *D. reticulatus* populations were characterized by a high prevalence of microorganism co-infections. 85.72–100.00% of the *D. reticulatus* ticks were infected by at least two microorganisms. There was no significant difference in the occurrence of co-infections between the examined sites (Fig. 3, Supplementary Table S1). A co-infection rate of 8.5% was reported in previous studies conducted in eastern Poland^60^. Polymicrobial infections by up to five pathogens were detected in *I. ricinus* ticks collected in the forest biotope, and 30.0–50.0% of ticks were not infected by any studied pathogen (Fig. 3). Co-infections of *I. ricinus* with TBPs has been also reported from other countries in Europe^61, 62^. In our opinion high rate of co-infections in examined tick species in current study is caused by favorable environmental conditions for both, hosts and ticks.

Our results show high prevalence of FLE infections in *D. reticulatus* ticks; regardless of the type of habitat they occupy (Tables 1 and 2; Supplementary Table S1). According to certain studies, co-infections with this microorganism affects immune system of ticks and promotes or limit infection of ticks by other pathogens, also affecting tick vector competence^63^.

The current study shows the genetic diversity of the *R. raoultii gltA* gene isolated from *D. reticulatus* ticks compared to other studies (Fig. 4). In addition, genetic differentiation of this gene was confirmed throughout the study period. In our opinion this is influenced by the type of biotopes. The meadow biotope proximity to the river causes seasonal floods, which can affect the structure of the tick population and their potential hosts; although adult *D. reticulatus* can survive temporal water flooding^64^. Similarly, periodical snowmelt floodings, occurring in early spring, were also observed in the forest biotope. Moreover, mowing and burning of meadows are other factors that can alter the size of populations of *D. reticulatus* and their hosts inhabiting such ecosystems. Such phenomena have been proven to reduce the abundance of ticks and thus can contribute to changes in the spectrum of vectored pathogens and their prevalence in ticks^65–68^.

## Conclusions

In overall, our study confirmed high prevalence of TBPs detected in sympatrically occurring adults of *I. ricinus* and *D. reticulatus* ticks, regardless of biotope type and climatic conditions. Substantial differences in spectrum of detected pathogens between the forest and meadow biotopes were observed in case of *N. mikurensis*, *Theileria* spp., Borreliaceae and Rickettsiaceae families. Moreover, our study confirmed significant impact of biotope type on prevalence of representatives of these families. Additionally, *Rickettsia* and *Anaplasma* temporal prevalence varied significantly across years. Studied tick populations in both biotopes were characterized by high co-infections rate. Our results also confirm significant diversity of *R. raoultii gltA* and *B. afzelii flaB* genes isolated in current study compared to reports from other countries. However, no relationship was found between the genetic diversity of the *R. raoultii gltA* gene isolated from examined ticks collected from the forest and the meadow biotopes. Additionally, temporal genetic diversity of *R. raoultii gltA* gene over the studied years was observed. Finally, our study confirmed impact of ecological type of biotope on spectrum and dynamic of pathogens prevalence in sympatrically occurring adult *I. ricinus* and *D. reticulatus* ticks.

## Materials and methods

### Ecological characteristic of study area

**Figure 5:**
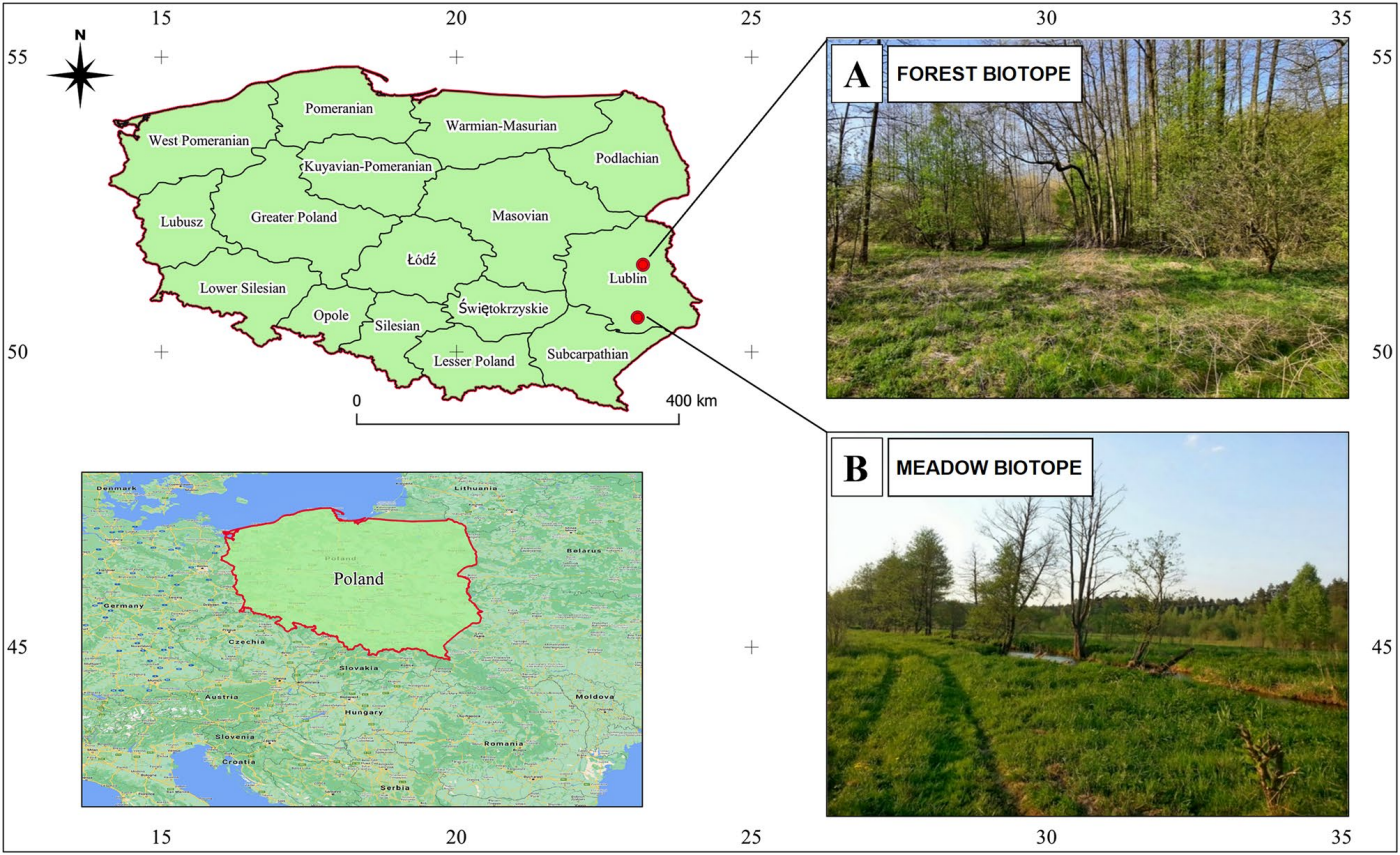
Tick collection sites. The map shows the localization of Poland and the studied areas on its map. A and B indicate the studied sites, i.e., forest and meadow biotopes, respectively.

The field study was conducted in eastern Poland. Ticks were collected in two different types of ecological biotopes, forest (A) and meadow (B) (Fig. 5). Selection of biotope locations to assess pathogen prevalence was based on long-term climatic data of the 10 years previous to the study, (see below), altitude, and potential natural vegetation (PNV)^69, 70^.

The study site established in the forest biotope (51.48° N 23.16° E) was located in a sub-region within a temperate climate with features of continentalism with an average annual air temperature of 9.2 °C, low annual rainfall of 550 mm and a short growing season (less than 200 days), and altitude of 200 m a.s.l. A large part of the region is characterized by agricultural wasteland (about 40%). The PNV represents the Querco-Pinetum, Carici elongatae-Alnetum, Peucedano-Pinetum and Tilio-Carpinetum^69–71^. The tick collection site was established in a clearing surrounded by deciduous forest with the dominance of Querco-Fagetea vegetation (Fig. 5). Such type of biotope is preferred by small rodents, mainly of the genera *Microtus* and *Apodemus*, which are the main hosts of juvenile stages of ticks^17, 72, 73^.

The meadow study site (50.585° N, 23.07° E) was located within a sub-region within the highest altitudes in eastern Poland (above 300 m.a.s.l.), where the mean annual temperature is 9.3 °C. Total annual precipitation reaches over 700 mm and is the highest value in the entire Lublin province. The landscape is characterized by the mosaic type of small arable fields often separated by mid-field scrubs. The sub-region is covered in 40% with forests dominated by plant species such as *Fagus sylvatica*, *Abies alba*, and *Picea abies*. The PNV belongs to Leucobryo-Pinetum, Querco-Pinetum, and Dentario glandulosae-Fagetum types^69–71^. The tick collection site was established in a fragment of an agriculturally unused meadow, with dominant Molinio-Arrhenatheretea vegetation, located in the immediate vicinity of the Wieprz River (Fig. 5).

### Tick collection and identification

The field study was carried out between 2018 and 2020 in eastern Poland. Collection was performed once per month, except in summer and winter months (July, August, November, December, January, and February) when behavioral diapause of *D. reticulatus* adults and substantial decrease of abundance of active adult stages of *I. ricinus* are observed^7, 17, 30^. To ensure an unbiased collection of ticks some criteria were followed: (*i*) questing ticks were collected systematically from the vegetation using the same method in all collections (i.e., flagging method^74^) following standard procedures (see below), (*ii*) ticks were invariably collected in the same transects of 900 m^2^ (30 × 30 m) in each site (i.e., one transect per site), (*iii*) tick collections were performed once per month in both sites, (*iv*) tick collections were synchronized (i.e., were carried out simultaneously at both study sites by different team members), and (*v*) ticks were collected only in days with similar weather conditions i.e., sunny, rainless and windless. The vegetation was swept with a 1-m^2^ white flannel cloth. After sweeping a transect of approximately 10-m, the cloth was turned over and attached ticks were transferred with metal tweezers into a 50-cm^3^ plastic container. This was repeated until the entire surface of the transects was covered. Grass blades were placed in the container to ensure the optimal level of humidity for the ticks. Additionally, each time when the ticks were collected, temperature and relative air humidity were measured at a level of 20 cm above the ground using a Data Loger device (R6030, Reed Instruments, Wilmington, NC, USA).

In the laboratory, the species, development stage, and sex of the tick specimens were identified using a Zeiss STEMI DV4 stereoscopic microscope (Carl Zeiss Light Microscopy, Göttingen, Germany). Taxonomically identification was based on morphological features and according to taxonomic key^74^. Next, the identified ticks were placed in an ULTF freezer (Arctico, Esbjerg, Denmark) at − 80 °C until DNA extraction.

Probably due to the ecological type of the studied habitats (high density of vegetation and tall grass), only a small number immature tick stages were collected. Therefore, only adult ticks of both species were included in further analyses.

### Long-term climatic data collection and selection of biotopes

In order to select biotope locations for our study, we investigated the differences in climatic conditions between two regions in eastern Poland, within which the study sites were established. To this end, long-term climatic data, covering 10-year period prior the field study (2008–2017) was collected and analyzed. Such data included mean monthly values of temperature (calculated on the basis of 8 measurements taken each day) and total precipitation, total monthly number of days with snow cover and total length of the vegetation season. Data was obtained from meteorological stations located closest to tick collection sites (about 20 km in a straight line), i.e., Włodawa station for the study site established in forest biotope and Tomaszów Lubelski station for the meadow biotope^71^.

## Molecular analyses

### DNA extraction

Prior to the genomic DNA extraction procedure, the tick bodies of randomly chosen 258 *D. reticulatus* and 185 *I. ricinus* specimens were cut into smaller fragments using sterile scalpels. The DNA was extracted using a NucleoSpin® Tissue DNA extraction kit (Macherey–Nagel, Düren, Germany) following the manufacturer's instructions. Ticks were homogenized using a Precellys 24 lyser/homogenizer (Bertin Technologies, Montigny-le-Bretonneux, France) at 5500 rpm for 20 s using 2.8 mm stainless steel beads in 180 µL of lysis buffer and 25 µL of Proteinase K from the Nucleospin Tissue kit (Macherey–Nagel, Düren, Germany). Homogenates were incubated for 3 h at 56 °C and DNA was extracted according to the manufacturer’s instructions. The concentration of DNA was measured using a NanoDrop™ spectrophotometer (Thermo Scientific, Waltham, Massachusetts, USA) at a 260/280 nm wavelength. The samples were stored at − 20 °C until further processing.

### DNA pre-amplification

The PreAmp Master Mix kit (Fluidigm, San Francisco, CA, USA) was used for DNA preamplification. The procedure was carried out in accordance with the manufacturer's recommendations. All primer pairs targeting TBPs were pooled, combining equal volumes with a final concentration of 0.2 µM each. The reaction was performed in a final volume of 5 μL containing 1 μL Perfecta Preamp 5 ×, 1.25 μL pooled primer mix, 1.5 µL distilled water and 1.25 μL DNA. The thermocycling program consisted of one cycle at 95 °C for 2 min, 14 cycles at 95 °C for 15 s and 4 min at 60 °C. The products were diluted 1:10 in Milli-Q ultrapure water and stored at − 20 °C until further use.

### Microfluidic real-time PCR for high-throughput microorganism detection

The BioMark™ real-time PCR system (Fluidigm, San Francisco, USA) was used for detection of the pathogens. Real-time PCR reactions were performed using 6-carboxyfluorescein (FAM)-labeled and black hole quencher (BHQ1)-labeled TaqMan probes with TaqMan Gene expression master mix according the manufacturer’s instructions (Applied Biosystems, France). The amplification process consisted of the following successive steps: 2 min at 50 °C, 10 min at 95 °C, followed by 40 cycles of two-step amplification of 15 s at 95 °C, and 1 min at 60 °C.

In the PCR reaction, 36 pathogen primers identical to those used by Boularias et al.^75^ were applied simultaneously. Additionally, primers targeting *I. ricinus* and *D. reticulatus* DNA were used to control for presence of tick species DNA. *Escherichia coli* DNA and *Escherichia*-specific primers were used as positive controls of the real-time PCR reactions. Ultra-pure water was used as a negative control. The real-time PCR results were analyzed using Fluidigm real-time PCR analysis software to obtain crossing threshold (Ct) values. Samples with a Ct value of < 25 was considered as positive.

### DNA sequencing

Samples positive for *Rickettsia* spp. and *Borreliella* spp. were sequenced due to the epidemiological importance of these pathogens among the microorganisms detected in the current study. For the sequence analysis, 3–5 samples of extracted DNA were selected from the genetic material obtained from each of the studied sites in 2018–2020 to identify pathogens of the genus *Rickettsia* and *Borreliella*. The PCR products were sequenced by Eurofins Genomics (https://cochin.eurofins.com, accessed on 5 August 2021). The sequenced products were analyzed using BioEdit software (Ibis Biosciences, Carlsbad, CA, USA).

### Phylogenetic analysis

To assess the genetic diversity of the microorganisms identified in this study, the obtained DNA sequences were analyzed using the Basic Local Alignment Search Tool (BLAST) with the NCBI database^76^ and similar sequences were searched in GenBank database^77^. Next, all sequences for specific pathogen and target genes available in BLAST were processed and redundant ones were deleted. Finally, phylogenetic trees were constructed with up to ten sequences obtained from other locations including Poland, Europe, Africa, Americas, Asia and Oceania, when available.

The evolutionary history was inferred by using the Maximum Likelihood method with complete deletion option and bootstrap set at 500 and analyzed in MEGA 11^78^. Depending on performed MEGA 11 analysis, different evolutionary models were used to construct the phylogenetic trees. Jukes-Cantor model was applied in case of *R. helvetica gltA, R. raoultii gltA* and *R. monacensis ompB*; while Kimura 2-parameter method was used in case of *B. afzelii flaB* and *B. garinii flaB*. Tamura 3-parameter model was chosen to construct *B. burgdorferi flaB* and *R. aeschlimannii gltA* phylogenetic tree. To determine genetic diversity of analyzed sequences, the proportion of nucleotide sites changes between them was calculated as p-distance in MEGA 11^78^.

## Statistical analysis

The type of data distribution was checked using the Shapiro–Wilk test. Long-term climatic data was tested using parametric t-test. Non parametric tests were used to check differences in the number of collected ticks between studied sites (U-Mann Whitney test), while the statistical differences in the value of temperature and humidity measured at the time of tick collection between studied sites over the years were tested using Kruskal–Wallis test. Spearman’s correlation coefficient test was used to determine association of temperature and humidity with tick activity.

The chi-square test was used to analyze the prevalence of microorganisms detected in *D. reticulatus* and *I. ricinus* ticks, as well as the differences between subsequent study years and biotopes. The significance of differences in p-distance values were compared using U-Mann Whitney test.

The significance level was set at *p* < 0.05. Statistical calculations were performed using the STATISTICA 13.3 PL statistical package (StatSoft, TIBCO Software Inc, Palo Alto, CA, USA) and GraphPad 8.4 (GraphPad Software Inc., La Jolla, CA, USA).

### Multiple correspondence analysis

Multiple correspondence analysis (MCA) was used to analyze the associations between the tick-borne pathogens, tick species and sampling sites (biotopes). The inertia values were calculated by the standard “Burt matrix” method. The analyses were performed using the statistical software package Statgraphics Centurion v. 16.1.03 (StatPoint Technologies Inc, Warrenton, VA, USA).

## Supplementary Information

Supplementary Information.

## Data Availability

All data generated in this study has been published in manuscript or Supplementary files.
