# Supplementary material for: Disparate dynamics of pathogen prevalence in Ixodes ricinus and Dermacentor reticulatus ticks occurring sympatrically in diverse habitats
Source: Sci Rep. 2023 Jun 30;13:10645. doi: 10.1038/s41598-023-37748-z (PMC10313804; doi:10.1038/s41598-023-37748-z)
Supplement: Supplementary file 1 — Supplementary Information. [file 41598_2023_37748_MOESM1_ESM.pdf]

Dynamics of tick-borne pathogens prevalence in sympatric tick species

Zbigniew Zając[1,*], Dasiel Obregon[2], Angélique Foucault-Simonin[3], Alejandra Wu-Chuang[3], Sara Moutailler[3], Clemence Galon[3], Joanna Kulisz[1], Aneta Woźniak[1], Katarzyna Bartosik[1], Alejandro Cabezas-Cruz[3,*]

[1] Department of Biology and Parasitology, Medical University of Lublin, Radziwiłłowska 11 st, 20-080, Lublin, Poland.
[2] School of Environmental Sciences University of Guelph, Guelph, Ontario N1G 2W1, Canada.
[3] Anses, INRAE, Ecole Nationale Vétérinaire d'Alfort, UMR BIPAR, Laboratoire de Santé Animale, 94700 Maisons-Alfort, France

***Correspondence:** A. Cabezas-Cruz (alejandro.cabezas@vet-alfort.fr); Zbigniew Zając (zbigniew.zajac@umlub.pl)

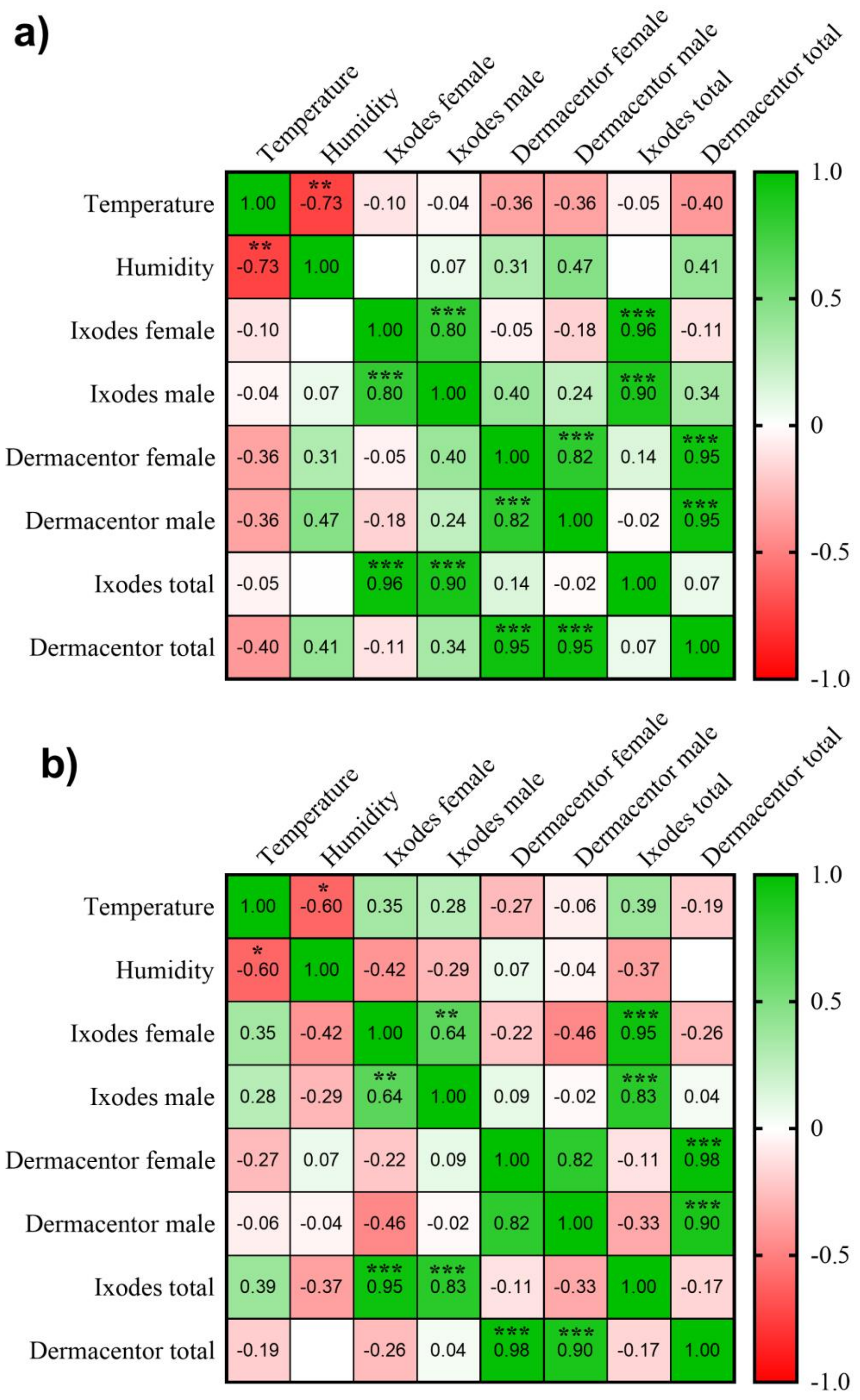

Supplementary Figure S1. Spearman rank correlograms on the association between environmental temperatures and relative humidity and the populations of *I. ricinus* and *D. reticulatus* ticks in the studied sites **a)** Forest biotope, and **b)** Meadow biotope. Negative and positive Spearman's correlation coefficient is indicated by cell colour gradient. Asterisks denote statistically significant correlations (* $p < 0.05$; ** $p < 0.01$; *** $p < 0.001$).

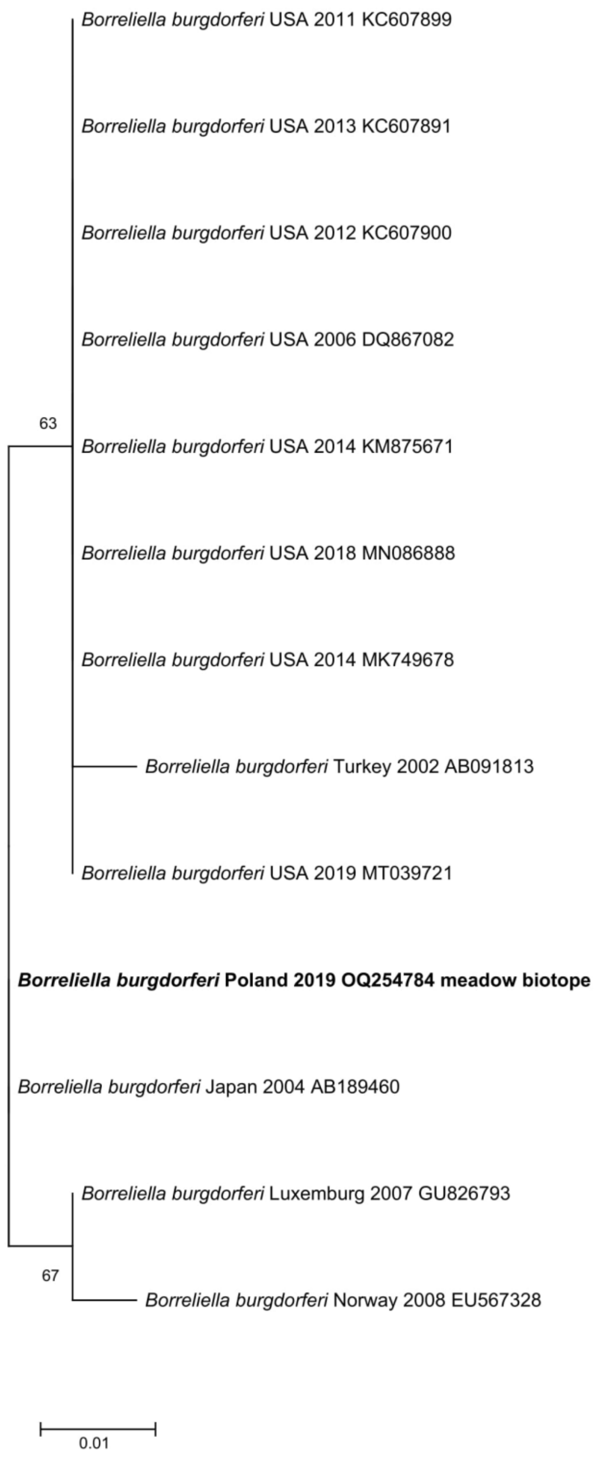

Supplementary Figure S2. Phylogenetic tree characterizing *Borreliella burgdorferi* s.s. *flaB* sequences. The evolutionary history was inferred by using the Maximum Likelihood method and the Tamura 3-parameter model. The analysis contains *B. burgdorferi flaB* sequences identified in studied *Ixodes ricinus* samples (marked with **bold**) and GenBank sequences. Accession numbers of sequences are given. Bootstrap values are represented as per cent of internal branches (500 replicates), values lower than 60 are hidden. The tree is drawn to scale, with branch lengths measured in the number of substitutions per site. All positions containing gaps and missing data were eliminated (complete deletion option). This analysis involved 13 nucleotide sequences. There was a total of 181 positions in the final dataset.

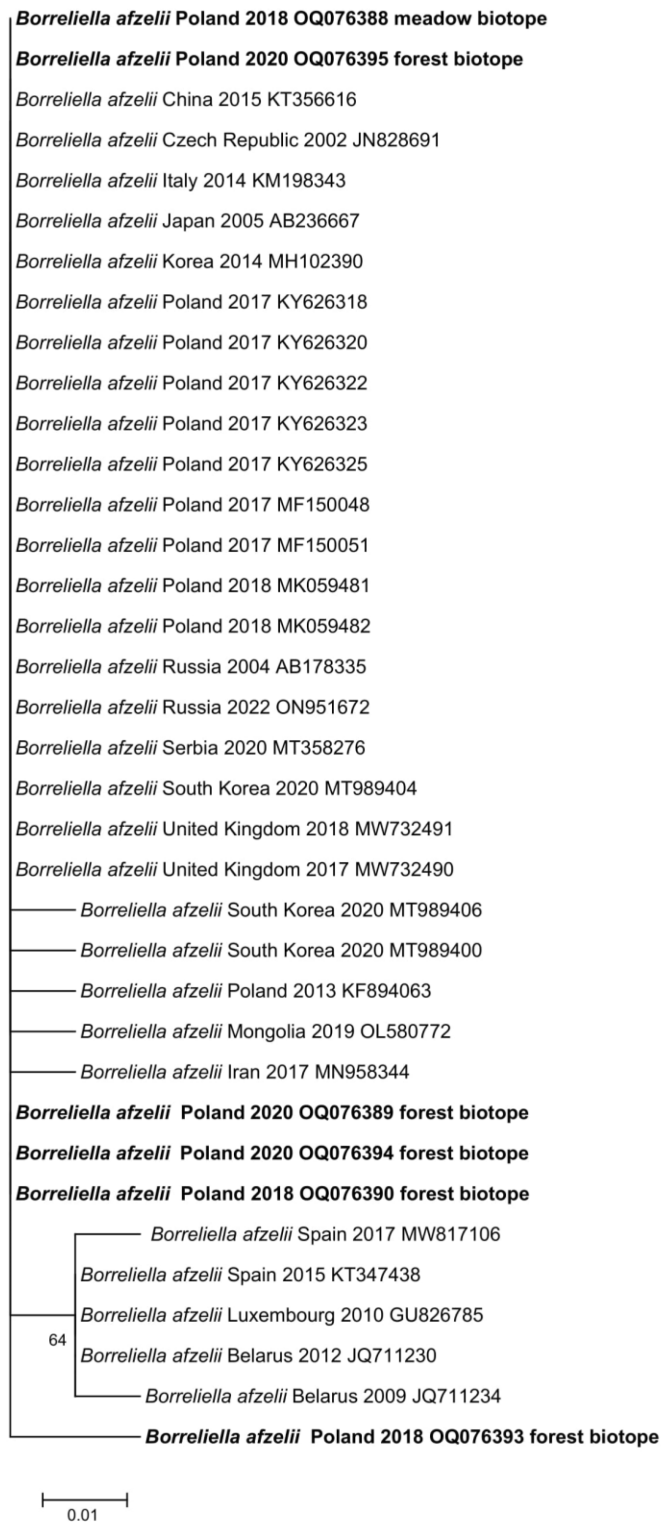

Supplementary Figure S3. Phylogenetic tree characterizing *Borreliella afzelii flaB* sequences. The evolutionary history was inferred by using the Maximum Likelihood method and the Kimura 2-parameter model. The analysis contains *B. afzelii flaB* sequences identified in studied *Ixodes ricinus* samples (marked with **bold**) and GenBank sequences. Accession numbers of sequences are given. Bootstrap values are represented as per cent of internal branches (500 replicates) values lower than 60 are hidden. The tree is drawn to scale, with branch lengths measured in the number of substitutions per site. This analysis involved 36 nucleotide sequences. All positions containing gaps and missing data were eliminated (complete deletion option). There was a total of 130 positions in the final dataset.

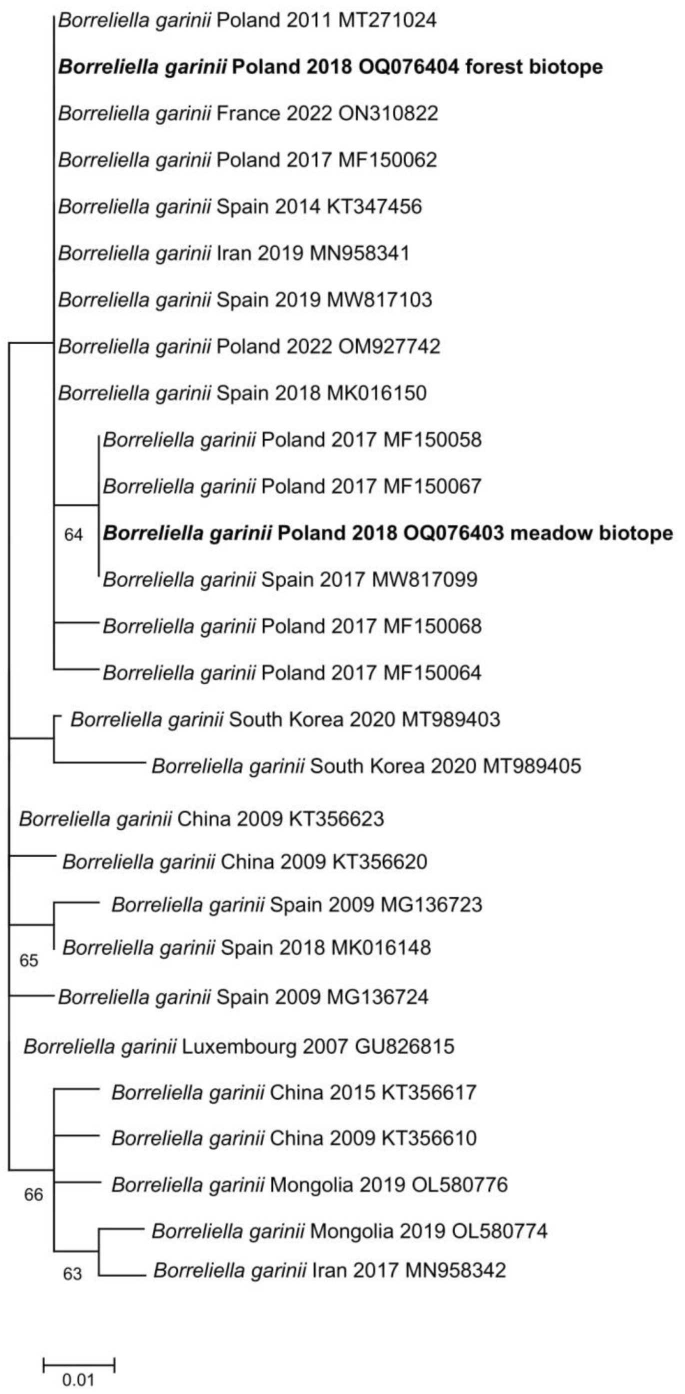

Supplementary Figure S4. Phylogenetic tree characterizing *Borreliella garinii flaB* sequences. The evolutionary history was inferred by using the Maximum Likelihood method and the Kimura 2-parameter model. The analysis contains *B. garinii flaB* sequences identified in studied *Ixodes ricinus* samples (marked with **bold**) and GenBank sequences. Accession numbers of sequences are given. Bootstrap values are represented as per cent of internal branches (500 replicates), values lower than 60 are hidden. The tree is drawn to scale, with branch lengths measured in the number of substitutions per site. This analysis involved 28 nucleotide sequences. All positions containing gaps and missing data were eliminated (complete deletion option). There was a total of 155 positions in the final dataset.

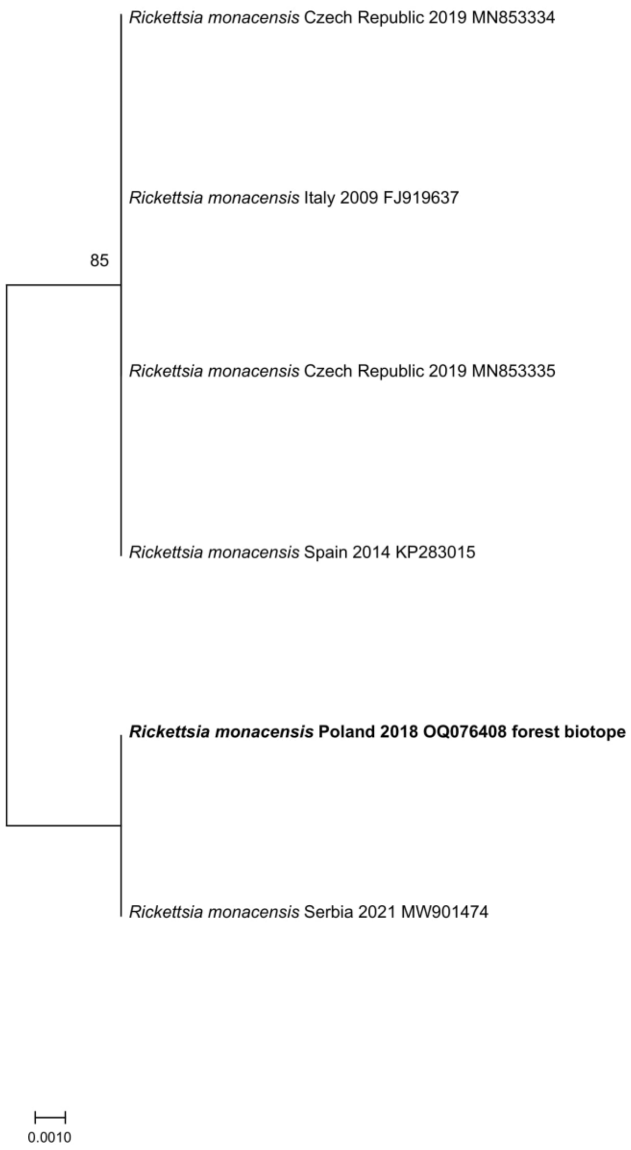

Supplementary Figure S5. Phylogenetic tree characterizing *Rickettsia monacensis ompB* sequences. The evolutionary history was inferred by using the Maximum Likelihood method and Jukes-Cantor model. Analysis contains *R. monacensis ompB* sequences identified in studied *Ixodes ricinus* samples (marked with **bold**) and GenBank sequences. Accession numbers of sequences are given. Bootstrap values are represented as percent of internal branches (500 replicates), values lower than 60 are hidden. The tree is drawn to scale, with branch lengths measured in the number of substitutions per site. This analysis involved 6 nucleotide sequences. All positions containing gaps and missing data were eliminated (complete deletion option). There was a total of 258 positions in the final dataset.

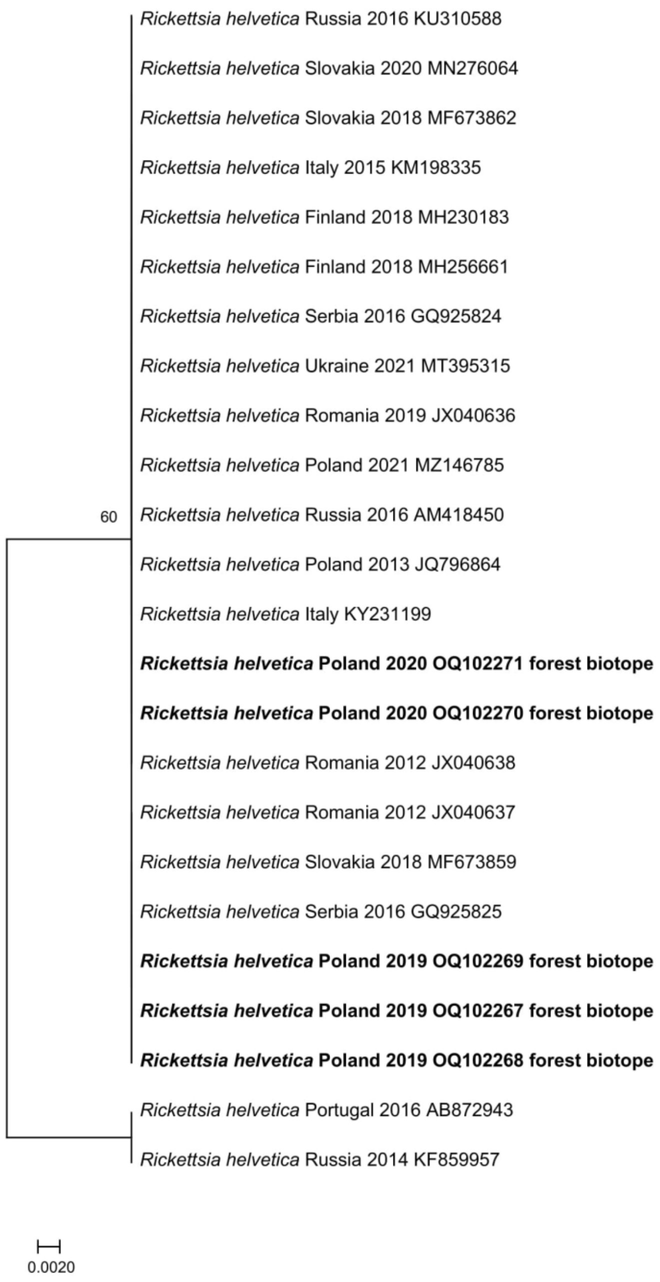

Supplementary Figure S6. Phylogenetic tree characterizing *Rickettsia helvetica gltA* sequences. The evolutionary history was inferred by using the Maximum Likelihood method and the Jukes-Cantor model. The analysis contains *R. helvetica* sequences identified in studied *Ixodes ricinus* samples (marked with **bold**) and GenBank sequences. Accession numbers of sequences are given. Bootstrap values are represented as per cent of internal branches (500 replicates), values lower than 60 are hidden. The tree is drawn to scale, with branch lengths measured in the number of substitutions per site. This analysis involved 14 nucleotide sequences. This analysis involved 24 nucleotide sequences. All positions containing gaps and missing data were eliminated (complete deletion option). There was a total of 46 positions in the final dataset.

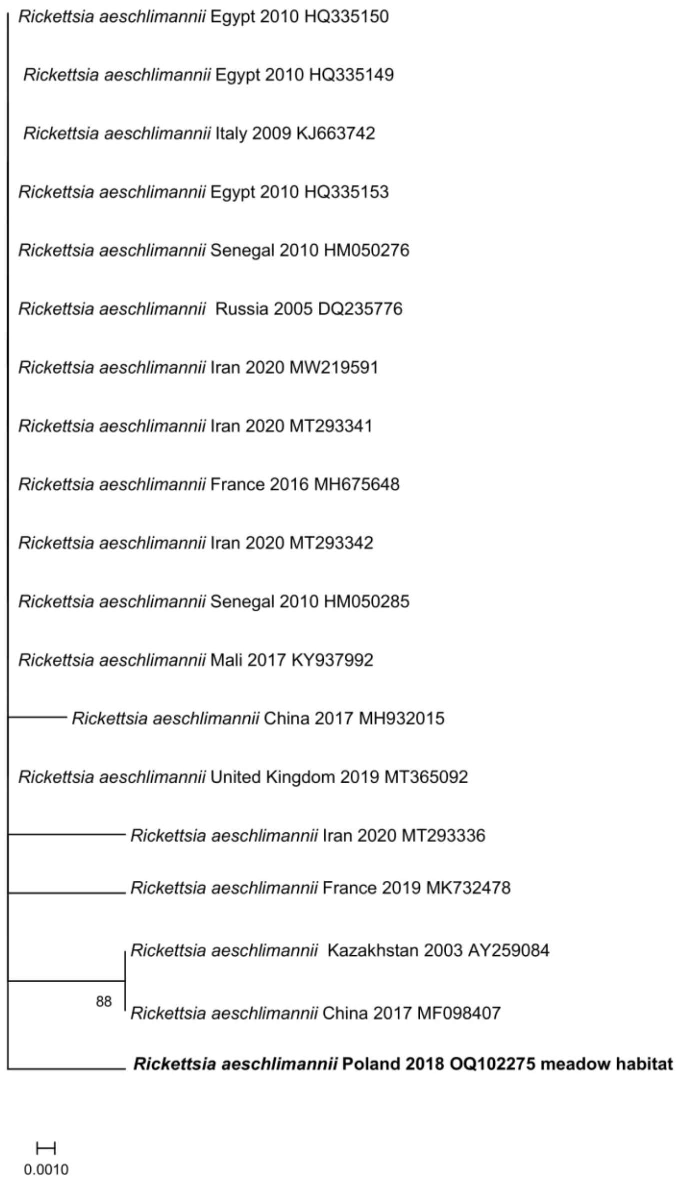

Supplementary Figure S7. Phylogenetic tree characterizing *Rickettsia aeschlimannii gltA* sequences. The evolutionary history was inferred by using the Maximum Likelihood method and the Tamura 3-parameter model. The analysis contains *R. aeschlimannii gltA* sequences identified in studied *Dermacentor reticulatus* samples (marked with **bold**) and GenBank sequences. Accession numbers of sequences are given. Bootstrap values are represented as per cent of internal branches (500 replicates), values lower than 60 are hidden. The tree is drawn to scale, with branch lengths measured in the number of substitutions per site. This analysis involved 19 nucleotide sequences. All positions containing gaps and missing data were eliminated (complete deletion option). There was a total of 305 positions in the final dataset.

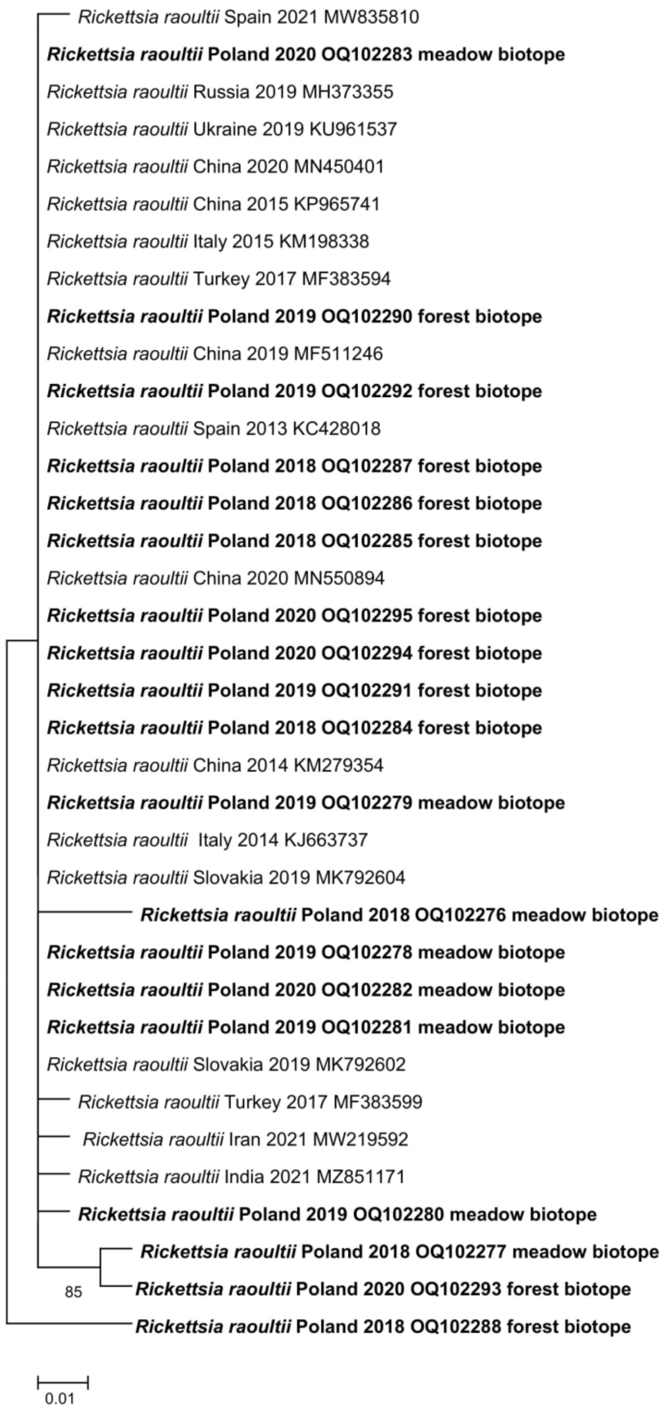

Supplementary Figure S8. Phylogenetic tree characterizing *Rickettsia raoultii gltA* sequences. The evolutionary history was inferred by using the Maximum Likelihood method and the Jukes-Cantor model. The analysis contains *R. raoultii gltA* sequences identified in studied *Dermacentor reticulatus* samples (marked with **bold**) and GenBank sequences. Accession numbers of sequences are given. Bootstrap values are represented as per cent of internal branches (500 replicates), values lower than 60 are hidden. The tree is drawn to scale, with branch lengths measured in the number of substitutions per site. This analysis involved 36 nucleotide sequences. All positions containing gaps and missing data were eliminated (complete deletion option). There was a total of 163 positions in the final dataset.

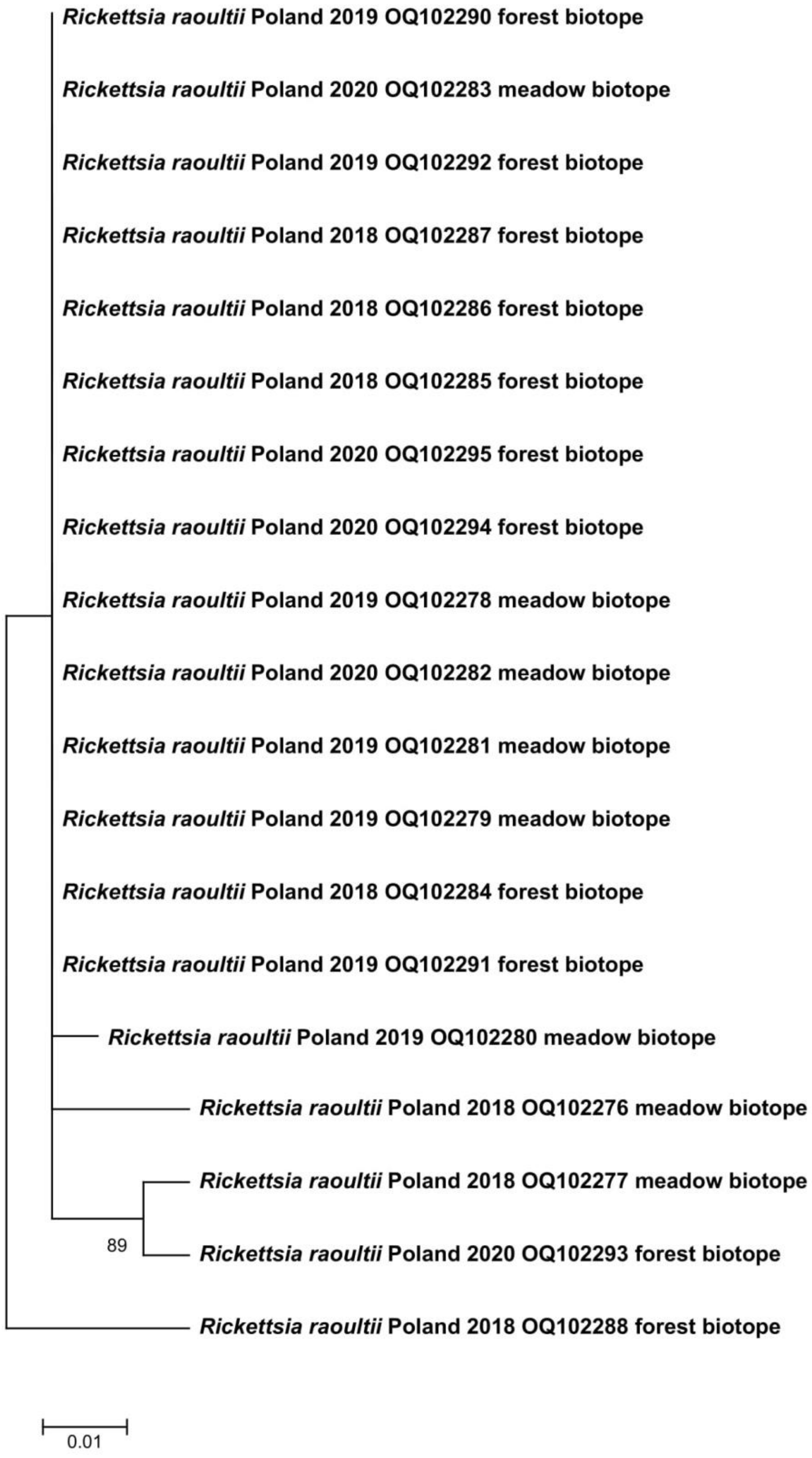

Supplementary Figure S9. Phylogenetic tree characterizing *Rickettsia raoultii gltA* sequences. The evolutionary history was inferred by using the Maximum Likelihood method and the Jukes-Cantor model. The analysis contains only *R. raoultii gltA* sequences identified in studied *Dermacentor reticulatus* samples (marked with **bold**). Accession numbers of sequences are given. Bootstrap values are represented as per cent of internal branches (500 replicates), values lower than 60 are hidden. The tree is drawn to scale, with branch lengths measured in the number of substitutions per site. This analysis involved 19 nucleotide sequences. All positions containing gaps and missing data were eliminated (complete deletion option). There was a total of 187 positions in the final dataset

Table S 1. Co-infections of microorganisms detected in *I. ricinus* and *D. reticulatus* in forest and meadow habitats (* - including possible coinfected ticks)

**Vector-borne pathogens**	**Number of infected ticks**	**Percentage rate [%]**
***Ixodes ricinus* Forest biotope 2018 n=20**		
**Total infected ticks (≥1 pathogen)***	**15**	**75.00**
Borreliella garinii	5	25.00
Borreliella afzelii	4	20.00
Borreliella spielmanii	1	5.00
Borrelia miyamotoi	1	5.00
Borreliella spp.	2	10.00
Anaplasma phagocytophilum	3	15.00
Anaplasma spp.	2	10.00
Ehrlichia spp.	2	10.00
Neoehrlichia mikurensis	1	5.00
Rickettsia monacensis	1	5.00
Rickettsia helvetica	7	35.00
Rickettsia amblyommatis	1	5.00
Rickettsia spp.	2	10.00
Bartonella henselae	2	10.00
Apicomplexa	3	15.00
**Single infections**	**4**	**20.00**
Borreliella spp.	1	5.00
Rickettsia spp.	1	5.00
Rickettsia amblyommatis	1	5.00
Apicomplexa	1	5.00
**Mixed infections**	**10**	**50.00**
**Mixed infections with two pathogens**	**5**	**25.00**
Borreliella spp. + *Rickettsia helvetica*	1	5.00
Borreliella spp. + *Borreliella garinii*	1	5.00
Anaplasma spp. + *Rickettsia* spp.	1	5.00
Borrelia miyamotoi + *Rickettsia helvetica*	1	5.00
Rickettsia helvetica + *Bartonella henselae*	1	5.00
**Mixed infections with three pathogens**	**1**	**5.00**
Borreliella garinii + *Rickettsia monacensis* + *Rickettsia amblyommatis*	1	5.00
**Mixed infections with four pathogens**	**1**	**5.00**
Borreliella garinii + *Borreliella afzelii* + *Borreliella spielmanii* + *Rickettsia helvetica*	1	5.00
**Mixed infections with five pathogens**	**3**	**15.00**
Borreliella garinii + *Borreliella afzelii* + *Ehrlichia* spp. *Anaplasma* spp.+ *Rickettsia helvetica*	1	5.00
Borreliella garinii + *Borreliella afzelii* + *Anaplasma* spp.+ *Rickettsia helvetica* + Apicomplexa	1	5.00
Borreliella afzelii + *Anaplasma* spp.+ *Anaplasma phagocytophilum*+ *Ehrlichia* spp. + *Rickettsia helvetica*	1	5.00

Ixodes ricinus Forest biotope 2019 n=64		
**Total infected ticks (≥1 pathogen)***	**39**	**60.94**
Borreliella afzelii	3	4.69
Borreliella lusitaniae	2	3.12
Borrelia miyamotoi	1	1.56
Borreliella spp.	3	4.69
Anaplasma phagocytophilum	5	7.81
Ehrlichia spp.	4	6.25
Neoehrlichia mikurensis	3	4.69
Rickettsia helvetica	23	35.94
Rickettsia spp.	5	7.81
Apicomplexa	7	10.94
Babesia venatorum	2	3.12
**Single infections**	**24**	**37.50**
Rickettsia spp.	4	6.25
Borreliella afzelii	1	1.56
Borreliella lusitaniae	1	1.56
Borreliella spp.	2	3.12
Anaplasma phagocytophilum	2	3.12
Ehrlichia spp.	1	1.56
Rickettsia helvetica	11	17.18
Apicomplexa	2	3.12
**Mixed infections**	**14**	**21.87**
**Mixed infections with two pathogens**	**10**	**15.62**
Borreliella afzelii + *Rickettsia* spp.	1	1.56
Borreliella lusitaniae + *Rickettsia helvetica*	1	1.56
Rickettsia helvetica + Apicomplexa	3	4.68
Borreliella afzelii + *Rickettsia helvetica*	1	1.56
Anaplasma phagocytophilum + *Rickettsia helvetica*	2	3.12
Borreliella spp. + *Rickettsia helvetica*	1	1.56
Ehrlichia spp. + *Neoehrlichia mikurensis*	1	1.56
**Mixed infections with three pathogens**	**2**	**3.12**
Rickettsia helvetica + Apicomplexa + *Babesia venatorum*	2	3.12
**Mixed infections with four pathogens**	**2**	**3.12**
Borrelia miyamotoi + *Ehrlichia* spp. + *Neoehrlichia mikurensis* + *Rickettsia helvetica*	1	1.56
Anaplasma phagocytophilum + *Ehrlichia* spp. + *Neoehrlichia mikurensis* + *Rickettsia helvetica*	1	1.56
Ixodes ricinus Forest biotope 2020 n=66		
**Total infected ticks (≥1 pathogen)***	**40**	**60.60**
Borreliella afzelii	4	6.06
Borreliella valaisiana	1	1.51
Borreliella spielmanii	1	1.51
Borrelia miyamotoi	2	3.03
Borreliella spp.	2	3.03
Anaplasma phagocytophilum	3	4.54

Ehrlichia spp.	1	1.51
Neoehrlichia mikurensis	1	1.51
Rickettsia helvetica	25	37.88
Apicomplexa	9	13.64
Babesia venatorum	3	4.54
**Single infections**	**30**	**45.45**
Rickettsia helvetica	19	28.78
Apicomplexa	3	4.54
Borrelia miyamotoi	1	1.51
Borreliella spielmanii	1	1.51
Borreliella spp.	2	3.03
Borreliella afzelii	2	3.03
Ehrlichia spp.	1	1.51
Anaplasma phagocytophilum	1	1.51
**Mixed infections**	**10**	**15.15**
**Mixed infections with two pathogens**	**8**	**12.12**
Borreliella valaisiana + *Rickettsia helvetica*	1	1.51
Anaplasma phagocytophilum + Apicomplexa	2	3.03
Borreliella afzelii + *Rickettsia helvetica*	2	3.03
Apicomplexa + *Babesia venatorum*	3	3.03
Borreliella spielmanii + *Rickettsia helvetica*	1	1.51
**Mixed infections with three pathogens**	**2**	**3.03**
Borrelia miyamotoi + *Rickettsia helvetica* + Apicomplexa	1	1.51
Rickettsia helvetica + Apicomplexa + *Babesia venatorum*	1	1.51
***Dermacentor reticulatus* Forest biotope 2018 n=24**		
**Total infected ticks (≥1 pathogen)***	**24**	**100.00**
Rickettsia spp.	22	91.67
Francisella-like endosymbiont	24	100.00
Apicomplexa	8	33.33
Babesia canis	3	12.5
**Single infections**	**2**	**8.33**
Francisella-like endosymbiont	2	8.33
**Mixed infections**	**22**	**91.66**
**Mixed infections with two pathogens**	**14**	**58.33**
Rickettsia spp. + *Francisella*-like endosymbiont	14	58.33
**Mixed infections with three pathogens**	**5**	**20.83**
Rickettsia spp. + *Francisella*-like endosymbiont + Apicomplexa	5	20.83
**Mixed infections with four pathogens**	**3**	**12.50**
Rickettsia spp. + *Francisella*-like endosymbiont + Apicomplexa + *Babesia canis*	3	12.50
***Dermacentor reticulatus* Forest biotope 2019 n=35**		

**Total infected ticks (≥1 pathogen)***	**35**	**100.00**
Anaplasma phagocytophilum	3	8.57
Rickettsia spp.	30	85.71
Francisella-like endosymbiont	35	100.00
Apicomplexa	7	20.00
Babesia canis	3	8.57
**Single infections**	**5**	**14.28**
Francisella-like endosymbiont	5	14.28
**Mixed infections**	**30**	**85.71**
**Mixed infections with two pathogens**	**19**	**54.28**
Rickettsia spp. + *Francisella*-like endosymbiont	19	54.28
**Mixed infections with three pathogens**	**9**	**25.71**
Rickettsia spp. + *Francisella*-like endosymbiont +*Babesia canis*	1	2.85
Anaplasma phagocytophilum + *Rickettsia* spp. + *Francisella-like* endosymbionts	2	5.71
Rickettsia spp. + *Francisella*-like endosymbiont + Apicomplexa	6	17.14
**Mixed infections with four pathogens**	**2**	**5.71**
Anaplasma phagocytophilum + *Rickettsia* spp. + *Francisella*-like endosymbiont + *Babesia canis*	1	2.85
Rickettsia spp. + *Francisella*-like endosymbiont + *Babesia canis* + Apicomplexa	1	2.85
Dermacentor reticulatus **Forest biotope 2020 n=22**		
**Total infected ticks (≥1 pathogen)***	**21**	**95.45**
Rickettsia helvetica	2	9.09
Rickettsia felis	1	4.54
Rickettsia spp.	15	68.18
Francisella-like endosymbiont	21	95.45
Apicomplexa	4	18.18
**Single infections**	**2**	**9.09**
Francisella-like endosymbiont	2	9.09
**Mixed infections**	**19**	**86.36**
**Mixed infections with two pathogens**	**15**	**68.18**
Rickettsia helvetica + *Francisella*-like endosymbiont	2	9.09
Rickettsia spp. + *Francisella*-like endosymbiont	11	50.00
Rickettsia felis + *Francisella*-like endosymbiont	1	4.54
Francisella-like endosymbiont + Apicomplexa	1	4.54
**Mixed infections with three pathogens**	**4**	**18.18**

Rickettsia spp. + *Francisella*-like endosymbiont + Apicomplexa	4	18.18
***Dermacentor reticulatus* Meadow biotope 2018 n=47**		
**Total infected ticks (≥1 pathogen)***	**46**	**97.87**
Rickettsia spp.	25	53.19
Rickettsia raoultii	3	6.38
Rickettsia aeschlimannii	1	2.13
Borreliella afzelii	1	2.13
Anaplasma phagocytophilum	6	12.77
Francisella-like endosymbiont	46	97.87
Apicomplexa	9	19.15
Babesia canis	2	4.26
Theileria spp.	1	2.13
**Single infections**	**11**	**23.40**
Rickettsia aeschlimannii	1	2.13
Francisella-like endosymbiont	10	21.28
**Mixed infections**	**34**	**72.34**
**Mixed infections with two pathogens**	**24**	**51.06**
Rickettsia spp. + *Francisella*-like endosymbiont	16	34.04
Anaplasma phagocytophilum + *Francisella*-like endosymbiont	3	6.38
Rickettsia raoultii + *Francisella*-like endosymbiont	2	4.26
Apicomplexa + *Francisella*-like endosymbiont	2	4.26
B. canis + *Francisella*-like endosymbiont	1	2.13
**Mixed infections with three pathogens**	**7**	**14.89**
Rickettsia spp. + *Francisella*-like endosymbiont + Apicomplexa	4	8.51
Rickettsia spp. + *Francisella*-like endosymbiont + *Borreliella afzelii*	1	2.13
Rickettsia spp. + *Francisella*-like endosymbiont + *Anaplasma phagocytophilum*	1	2.13
Rickettsia raoultii + *Francisella*-like endosymbiont + *Anaplasma phagocytophilum*	1	2.13
**Mixed infections with four pathogens**	**3**	**6.38**
Rickettsia + *Francisella*-like endosymbiont + Apicomplexa + *Theileria* spp.	1	2.13
Rickettsia + *Francisella*-like endosymbiont + Apicomplexa + *B. canis*	1	2.13
Rickettsia + *Francisella*-like endosymbiont + Apicomplexa + *Anaplasma phagocytophilum*	1	2.13
***Dermacentor reticulatus* Meadow biotope 2019 n=72**		
**Total infected ticks (≥1 pathogen)***	**66**	**91.67**
Rickettsia spp.	47	65.28
Rickettsia raoultii	4	5.56
Anaplasma phagocytophilum	4	5.56
Francisella-like endosymbiont	62	86.11

Apicomplexa	10	13.89
Babesia canis	2	2.78
**Single infections**	**15**	**20.83**
Rickettsia spp.	3	4.16
Francisella-like endosymbiont	12	16.67
**Mixed infections**	**51**	**70.83**
**Mixed infections with two pathogens**	**39**	**54.16**
Rickettsia spp. + *Francisella*-like endosymbiont	33	45.83
Rickettsia spp. + *Anaplasma phagocytophilum*	1	1.38
Rickettsia raoultii + *Francisella*-like endosymbiont	2	2.78
Francisella-like endosymbiont+ Apicomplexa	2	2.78
Anaplasma phagocytophilum + *Francisella*-like endosymbiont	1	1.38
**Mixed infections with three pathogens**	**12**	**16.67**
Rickettsia spp. + *Francisella*-like endosymbiont + Apicomplexa	8	11.11
Anaplasma phagocytophilum + *Rickettsia* spp. + *Francisella*-like endosymbiont	2	2.78
Rickettsia spp. + *Francisella*-like endosymbiont+ *B. canis*	1	1.38
Rickettsia raoultii + *Francisella*-like endosymbiont+ *B. canis*	1	1.38
***Dermacentor reticulatus* Meadow biotope 2020 n=58**		
**Total infected ticks (≥1 pathogen)***	**58**	**100.00**
Anaplasma phagocytophilum	5	8.62
Anaplasma spp.	1	1.72
Ehrlichia spp.	1	1.72
Rickettsia raoultii	2	3.44
Rickettsia spp.	44	75.56
Bartonella spp.	2	3.44
Francisella-like endosymbiont	55	94.83
Apicomplexa	8	13.79
Babesia canis	2	3.44
**Single infections**	**11**	**18.96**
Rickettsia spp.	3	5.17
Francisella-like endosymbiont	8	13.79
**Mixed infections**	**47**	**81.03**
**Mixed infections with two pathogens**	**35**	**60.34**
Rickettsia spp.+ *Francisella*-like endosymbiont	30	51.72
Francisella-like endosymbiont+ Apicomplexa	2	3.44
Ehrlichia spp.+ *Francisella*-like endosymbiont	1	1.72
Anaplasma phagocytophilum+ *Francisella*-like endosymbiont	1	1.72

Rickettsia raoultii+ *Francisella*-like endosymbiont	1	1.72
**Mixed infections with three pathogens**	**9**	**15.51**
Rickettsia spp.+ *Francisella*-like endosymbiont+ Apicomplexa	4	6.89
Rickettsia spp.+ *Bartonella* spp.+ *Francisella*-like endosymbiont	1	1.72
Anaplasma phagocytophilum+ *Rickettsia* spp.+ *Francisella*-like endosymbiont	2	3.44
Anaplasma spp.+ *Rickettsia* spp.+ *Francisella*-like endosymbiont	1	1.72
Rickettsia raoultii + *Francisella*-like endosymbiont+ Apicomplexa	1	1.72
**Mixed infections with three pathogens**	**3**	**5.17**
Anaplasma phagocytophilum+ *Rickettsia* spp.+ *Francisella*-like endosymbiont+*B. canis*	1	1.72
Anaplasma spp.+ *Rickettsia* spp.+ *Francisella*-like endosymbiont+ *Bartonella* spp.	1	1.72
Rickettsia spp.+ *Francisella*-like endosymbiont+ Apicomplexa + *B. canis*	1	1.72
Ixodes ricinus **Meadow biotope 2018 n=15**		
**Total infected ticks (≥1 pathogen)***	**10**	**66.66**
Borreliella burgdorferi s.s.	2	13.33
Borreliella garinii	3	20.00
Borreliella afzelii	1	6.67
Borreliella spielmanii	2	13.33
Anaplasma phagocytophilum	4	26.67
Rickettsia spp.	2	13.33
Francisella-like endosymbiont	1	6.67
Apicomplexa	3	20.00
**Single infections**	**4**	**26.67**
Anaplasma phagocytophilum	1	6.67
Apicomplexa	3	20.00
**Mixed infections**	**6**	**40.00**
**Mixed infections with two pathogens**	**4**	**26.67**
Borreliella burgdorferi + *Borreliella garinii*	1	6.67
Anaplasma phagocytophilum + *Rickettsia* spp.	2	13.33
Rickettsia spp. + *Francisella*-like endosymbiont	1	6.67
Borreliella spielmanii + *Anaplasma phagocytophilum*	1	6.67
**Mixed infections with three pathogens**	**2**	**13.33**
Borreliella garinii + *Borreliella spielmanii* + *Borreliella afzelii*	1	6.67
Borreliella burgdorferi + *Borreliella garinii* + *Anaplasma phagocytophilum*	1	6.67
Ixodes ricinus **Meadow biotope 2019 n=12**		
**Total infected ticks (≥1 pathogen)***	**7**	**58.33**

Borreliella spp.	2	16.67
Borreliella burgdorferi	2	16.67
Bartonella spp.	2	16.67
Anaplasma phagocytophilum	1	8.33
Ehrlichia spp.	1	8.33
Borreliella valaisiana	1	8.33
Borrelia miyamotoi	1	8.33
**Single infections**	**4**	**33.33**
Borreliella spp.	1	8.33
Borreliella burgdorferi	1	8.33
Bartonella spp.	1	8.33
Borreliella valaisiana	1	8.33
**Mixed infections**	**3**	**25.00**
**Mixed infections with two pathogens**	**3**	**25.00**
Anaplasma phagocytophilum + *Bartonella* spp.	1	8.33
Borreliella burgdorferii + *Borrelia miyamotoi*	1	8.33
Borreliella spp. + *Ehrlichia* spp.	1	8.33
Ixodes ricinus **Meadow biotope 2020 n=8**		
**Total infected ticks (≥1 pathogen)***	**4**	**50.00**
Borreliella garinii	1	12.50
Borreliella afzelii	1	12.50
Borreliella spielmanii	1	12.50
Borreliella spp.	1	12.50
Rickettsia spp.	1	12.50
Bartonella henselae	1	12.50
**Single infections**	**3**	**37.50**
Bartonella henselae	1	12.50
Rickettsia spp.	1	12.50
Borreliella spp.	1	12.50
**Mixed infections**	**1**	**12.50**
**Mixed infections with three pathogens**	1	12.50
Borreliella garinii + *Borreliella afzelii* + *Borreliella spielmanii*	1	12.50
